# A novel TAp73‐inhibitory compound counteracts stemness features of glioblastoma stem cells

**DOI:** 10.1002/1878-0261.13694

**Published:** 2024-08-01

**Authors:** Javier Villoch‐Fernandez, Nicole Martínez‐García, Marta Martín‐López, Laura Maeso‐Alonso, Lorena López‐Ferreras, Alberto Vazquez‐Jimenez, Lisandra Muñoz‐Hidalgo, Noemí Garcia‐Romero, Jose María Sanchez, Antonio Fernandez, Angel Ayuso‐Sacido, Margarita M. Marques, Maria C. Marin

**Affiliations:** ^1^ Instituto de Biomedicina y Departamento de Biología Molecular Universidad de León Spain; ^2^ Instituto de Biomedicina y Departamento de Producción Animal Universidad de León Spain; ^3^ Biomar Microbial Technologies, Parque Tecnológico de León Spain; ^4^ Department of Pathology, Faculty of Medicine and Odontology Universidad de Valencia Spain; ^5^ Faculty of Experimental Sciences Universidad Francisco de Vitoria Madrid Spain; ^6^ Brain Tumor Laboratory, Fundación Vithas Grupo Hospitales Vithas Madrid Spain; ^7^ Faculty of Medicine Universidad Francisco de Vitoria Madrid Spain; ^8^ Instituto de Desarrollo Ganadero y Sanidad Animal y Departamento de Producción Animal Universidad de León Spain

**Keywords:** glioblastoma, glioblastoma stem cells, natural compound, stemness signature, TAp73

## Abstract

Glioblastoma (GB) is the most common and fatal type of primary malignant brain tumor for which effective therapeutics are still lacking. GB stem cells, with tumor‐initiating and self‐renewal capacity, are mostly responsible for GB malignancy, representing a crucial target for therapies. The *TP73* gene, which is highly expressed in GB, gives rise to the TAp73 isoform, a pleiotropic protein that regulates neural stem cell biology; however, its role in cancer has been highly controversial. We inactivated *TP73* in human GB stem cells and revealed that TAp73 is required for their stemness potential, acting as a regulator of the transcriptional stemness signatures, highlighting TAp73 as a possible therapeutic target. As proof of concept, we identified a novel natural compound with TAp73‐inhibitory capacity, which was highly effective against GB stem cells. The treatment reduced GB stem cell‐invasion capacity and stem features, at least in part by TAp73 repression. Our data are consistent with a novel paradigm in which hijacking of p73‐regulated neurodevelopmental programs, including neural stemness, might sustain tumor progression, pointing out TAp73 as a therapeutic strategy for GB.

## Graphical abstract

In glioblastoma (GB) tumors, a dynamic population of cancer stem cells (CSCs) drives tumor initiation, resistance, and recurrence. In this work, we demonstrated that TAp73 is crucial to sustain CSC features. We identified a TAp73‐repressive natural compound (BMT9) that impairs key GB stem cell properties. Together, our results postulate *TP73* as an interesting therapeutic target, particularly in advanced tumor stages.

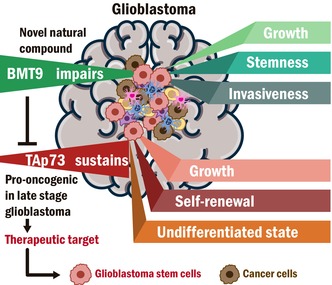

**Abbreviations**
BCNU: carmustine
CNS: central nervous system
CSCs: cancer stem cells
DEGs: differentially expressed genes
FDR: false discovery rate
GB: glioblastoma
GO: gene ontology
GSCs: stem‐like glioblastoma cells
GSEA: gene set enrichment analysis
HEF: human embryonic fibroblast
NS: neurosphere
NSCs: neural stem cells
p73KO: *TP73* knockout
P‐His3: phospho‐histone 3
SEM: standard error of the mean
SVZ: subventricular zone
TG: thapsigargin
TMZ: temozolomide

## 1 Introduction

Adult‐type diffuse gliomas, which comprises glioblastoma (GB), IDH wild‐type, is the most common and aggressive form of glial tumors, accounting for almost 50% of primary malignant central nervous system (CNS) tumors [1]. These tumors arise *de novo* and typically manifest rapidly after a short clinical history [2]. Although the overall estimates of survival have improved during the last decades due to a multimodal therapeutic approach (including surgery, radiotherapy, and chemotherapy), long‐term survival remains poor with almost no increase in 5‐year survival [3]. The absence of proper treatments, together with GB invasiveness and increasing incidence, have sparked the search for new therapeutic targets. In order to design new strategies to find effective therapies, it is important to understand the origin and progression of GB. In this regard, several studies have suggested that GB are sustained from a subsection of cells called stem‐like glioblastoma cells (GSCs) that act as a reservoir for tumor regeneration; however, identifying the cell of origin harboring the mutations that generate these GSCs has been elusive. Several studies have suggested that adult neural stem cells (NSCs) may be the cells from which GSCs originate, based on the fact that GSCs and NSC share many common features, including expression of stem and progenitor cell markers (CD133/Prominin‐1, among others), and self‐renewal and multilineage generation capacity [4]. Indeed, recent studies have demonstrated that astrocyte‐like NSCs within the subventricular zone (SVZ) of the adult human brain are the cells of origin that contain the driver mutations of human GB [5]. While this origin of primary GB was confirmed, other authors suggested that GB originates from outer radial glial cells [6]. Thus, a ‘double origin’ hypothesis of GB from a mixed population of ventricular and outer radial glial cells in SVZ is nowadays under debate [7, 8]. Independently of their cell of origin, GSCs are believed to be responsible, at least in part, for the GB initiation, progression, heterogeneity, recurrency, and increased resistance to treatments [9, 10, 11]. These features make GSCs a very attractive model to test candidate compounds to treat GB. As such, regulators of the stemness networks that sustain cancer stem cells (CSCs), are therapeutically pursued. In this regard, several publications have shown that the *TP73* gene, which belongs to the p53 family and has been commonly regarded as a putative tumor suppressor, may positively regulate the growth and stemness of CSCs [12, 13, 14], therefore playing a non‐expected role in stem cell biology. This postulates *TP73* as an interesting therapeutic target.

The *TP73* gene can give rise to different isoforms: TAp73 with a full transactivation domain, and the amino‐terminally truncated DNp73 isoforms [15]. Unlike *TP53*, a bona fide tumor suppressor gene, which is mutated in 50% of human tumors [16, 17, 18, 19], a great body of work has revealed that *TP73* is rarely mutated in cancer [20, 21, 22, 23, 24, 25]. On the contrary, high levels of TAp73 have been detected in multiple types of tumors, such as cervical carcinoma [26], B‐Cell chronic lymphocytic leukemia [27], ovarian carcinoma [28], gastric adenocarcinoma [29], medulloblastoma [30], and GB [31]. In addition, TAp73 expression sustains cell growth through the regulation of glutamine metabolism and TAp73 silencing leads to glutamine starvation in medulloblastoma cells, reducing cell growth, oxygen consumption, and basal respiration [32]. Furthermore, TAp73 cooperates with several AP‐1 members to sustain cellular proliferation [33, 34] and activates anabolic pathways, all compatible with proliferation and promotion of cancer cells [35, 36, 37].

The role of TAp73 in brain biology is of particular relevance since p73 is essential not only for the embryonic development of the murine CNS [38, 39], but also for the organization and maintenance of murine NSCs niches [40]. Indeed, TAp73 is required to maintain NSC stemness and p73‐deficiency results in premature differentiation of NSCs [41, 42]. As stated before, GSCs have similarities to NSCs [43], and in accordance, TAp73 positively regulates growth and self‐renewal of CD133+ patient‐derived brain tumor‐initiating cells [14], all backing up TAp73 role in sustaining GSCs maintenance and GB progression. Thus, treatments that modulate or repress p73 isoforms could be of interest to manage p73‐overexpressing tumors.

In this work, we revealed *TP73* as a transcriptional regulator of stemness gene signatures and confirmed p73 requirement to maintain human GSC stemness, marking the TAp73 isoform as an interesting therapeutic target. Using a low‐throughput screening system, aimed to discover regulators of the *TP73*‐P1‐promoter, we identified a novel natural compound with TAp73 inhibitory capacity. This compound shows high effectiveness against GSCs, strongly reducing key CSC features, such as invasiveness capacity and stemness potential.

## 2 Materials and methods

### 2.1 Cell culture

Human GSC lines (G144‐CVCL_DG64 and G179‐CVCL_DG69) and human fetal NSCs (FT430, derived from the forebrain) were kindly provided by S. M. Pollard (Centre for Regenerative Medicine and Edinburgh Cancer Research Centre, University of Edinburgh). The GSC cell lines were characterized for their stem cell properties (sphere forming assays, evaluation of differentiation properties, and marker expression) and cultured under adherent conditions in serum‐free media supplemented with N2, B27, EGF, and FGF‐2 as described previously [44]. mEGF, hFGF (20 ng·mL^−1^ each; Peprotech, Cranbury, NJ, USA), and laminin (10 μg·mL^−1^; Sigma‐Aldrich, St. Louis, MO, USA) were freshly added to the medium prior to each use. The G144 and G179 cell lines were derived from male adult patients (51‐ and 56 years old, respectively), who were diagnosed with a classic GB multiform (G144) and a giant cell variant (G179) [44]. Both cell lines are IDH1 wild‐type [45] and carry p53 mutations; in particular, the G144 cells contain a mutant p53 protein (Pro152Leu) [46]. GBM18 and GBM27 GSCs‐enriched cell lines were derived from fresh surgical patient GB samples by N. García‐Romero at Dr Ayuso's laboratory (Brain Tumor Laboratory, Grupo Hospitales Vithas, Madrid), and cultured as described previously [47]. All cell lines were authenticated by STR‐DNA profiling in the past 3 years.

Conventional GB cell lines (T98G‐CVCL_0556 and U373‐CVCL_2818), as well as the primary GB cell line H3444 (GB‐val4, CVCL_XZ67) (kindly provided by Dra. López Ginés [48]), were cultured in RPMI (T98G and H3444) or in DMEM (U373) supplemented with 10% fetal bovine serum (FBS; Sigma‐Aldrich), 2 mm l‐Glutamine (Sigma‐Aldrich), 1 mm non‐essential amino acids (Gibco, Waltham, MA, USA), and 1 mm Sodium Pyruvate (Gibco). HCT116p53−/− cells were maintained in RPMI (Sigma‐Aldrich) supplemented with 10% FBS and 2 mm l‐Glutamine.

All cell lines used in this study were periodically tested to ensure that they were mycoplasma‐free using the Mycoplasma Gel Form Kit (Biotools B & M Labs, Villaverde, Madrid, Spain). GSC cell lines were routinely authenticated by GSC marker expression analysis. Specifically, CD133 expression (quantified by flow cytometry) and Nestin expression (evaluated by immunofluorescence and confocal microscopy) were assessed.

For the neurosphere formation assay, recommendations by Belenguer et al. [49] were followed to avoid cell aggregation and obtain a reproducible and reliable quantification. G144 cells were seeded at clonal concentration (4000 cells·mL^−1^) in routine G144 cell growth medium without laminin, to achieve suspension culture. After 8 days, spheres were counted, dissociated with Accutase solution (Sigma‐Aldrich), and seeded under similar conditions for up to three passages. Spheres were counted manually. Briefly, triplicate samples (50 μL) of the sphere suspension were placed in a 96‐well plate, and sphere number was counted using a Leica DMI3000 phase‐contrast microscope (Leica Microsystems CMS GmbH, Wetzlar, Germany) coupled to a digital camera LEICA DFC 310FX. The total number of spheres·mL^−1^ per condition was extrapolated from that data. Only spherical spheres with more than 50 μm diameter were considered, while irregular clumps were discarded as they could be formed by cell aggregation.

For cell morphology analysis, G144 lines were seeded at low cell density (1250 cell·cm^−2^). After 24 h, phase‐contrast images were acquired every 5 min at 37 °C, 5% CO_2_ on a Zeiss LSM800 Confocal microscope (Carl Zeiss Industrielle Messtechnik GmbH, Oberkochen, Germany) using an ESID Detector Module. At least 70 cells per condition were manually categorized based on the criteria of showing a polarized morphology (reminiscent of fetal NSCs) or a flat apolar morphology [43, 44, 50].

### 2.2 Inactivation of the *TP73* gene in G144 cells using the CRISPR/Cas9 gene editing system

To generate the G144‐p73 knockout cell lines, we targeted Exon 8 of the *TP73* gene by CRISPR/Cas9 gene editing. A single‐guide RNA (sgRNA) was designed (5′‐AACGGGGCCGCCAGCAAGCGTGG‐3′) using the webtool CHOPCHOP [51] and cloned in the sgRNA scaffold of the PX459 plasmid (#62988; Addgene, Watertown, MA, USA), to obtain the PX459p73Ex8 plasmid. Then, G144 cells were nucleofected (3 × 10^6^ cells) using an Amaxa Nucleofector II (Lonza, Basel, Switzerland) and the Nucleofection kit V (Ref: VCA‐1003; Lonza) with the T‐020 program. Twenty‐four hours after transfection, puromycin selection was applied (2 μg·mL^−1^) for 5 days, and the selected cells were replated at clonal conditions in 96‐well plates. Several colonies were isolated and expanded for genotyping. Genomic DNA was extracted by incubating the cells with lysis buffer (100 mm Tris–HCl pH 8.5, 200 mm NaCl, 5 mm EDTA, 0.2% SDS, 10 mg·mL^−1^ Proteinase K) overnight at 65 °C, followed by DNA precipitation with isopropanol. Gene editing was analyzed by amplifying a region of 242 bp using the following primers: Fw: 5′‐AAAGCTGATGAGGACCACTA‐3′ and Rv 5′‐GACTTATATGGGGGCTGCTC‐3′. PCR products were resolved in a 1% agarose gel and sequenced at the Nucleic Acid Analysis Service from Universidad de León. One clone that was subjected to the same gene editing procedure (nucleofection with the PX459p73Ex8 vector, followed by puromycin treatment and clonal isolation), but that did not result in a change in sequence, was selected as *TP73* non‐edited control. To confirm gene inactivation at RNA level, a qRT‐PCR analysis was performed in the selected clones (G144‐KO1 and G144‐KO2), using a pair of primers that match the target sequence (Fw 5′‐ACCGAAAAGCTGATGAGGAC‐3′, Rv 5′‐CTTGAAGGCACGCTTGC‐3′).

### 2.3 Drug screening

The P1‐p73‐Fluc‐DsRed reporter vector was generated by swapping the CMV promoter for the P1‐*TP73* promoter in the bimodal vector LV‐CMV‐Fluc‐DsRed, kindly provided by K. Shah (Harvard Medical School, Boston, USA) [52]. A 930‐bp sequence of the human P1‐*TP73* promoter, spanning from −859 to +71, was subcloned from the P1‐p73‐Fluc‐pGL3‐Basic vector [53], into the LV‐CMV‐Fluc‐DsRed plasmid.

For the screening, U373 cells were transfected with the reporter vector, using Lipofectamine™ 3000 reagent (Invitrogen, Waltham, MA, USA) following the manufacturer's instructions. The transfection mixture was replaced 6 h after transfection with medium containing the tested compounds. The compounds for the low‐throughput screening (BMT1 to BMT51) were produced by Biomar Microbial Technologies (León, Spain) and resuspended in DMSO, at a stock concentration of 10 mg·mL^−1^. Temozolomide (TMZ) and Carmustine (BCNU) were purchased from Sigma‐Aldrich (Ref: T2577 and C0400, respectively), and resuspended in DMSO at 100 and 1000 mm, respectively. The DsRed reporter expression was monitored by fluorescence microscopy (Zeiss LSM800 Confocal Laser Scanning Microscope) and quantified by fluorimetry (Synergy HT plate reader; Biotek, Agilent Technologies, Santa Clara, CA, USA) 48 h after treatment.

### 2.4 Cell viability assays

Cell viability was evaluated using the MTT‐based cytotoxicity assay. Briefly, cells were seeded in 96‐well plates at the following cell densities: G144, G179, and NSC: 62 500 cells·cm^−2^; T98G: 3200 cells·cm^−2^; U373: 6400 cells·cm^−2^; H3444: 3200 cells·cm^−2^. Twenty‐four hours later, they were treated in triplicates with the different compounds. Controls contained the DMSO volume equivalent to the highest volume used for the treatments and never exceeded 0.001% (see details in figure legends). At the indicated time points, the MTT tetrazolium salt (Sigma‐Aldrich) diluted in 1× PBS was added to each well to a final concentration of 0.33 mg·mL^−1^ and incubated for 3 h at 37 °C. Then, the media was removed, and formazan crystals were solubilized with DMSO. Absorbance was measured with a Synergy HT plate reader (Biotek, Agilent Technologies) at 560 nm. In the case of GBM18 and GBM27 cell lines, cell viability was quantified using the MTS‐based cytotoxicity with the CellTiter 96® Aqueous One Solution Cell Proliferation Assay kit (Promega, Madison, WI, USA). Briefly, cells were seeded in suspension at a cell concentration of 40 000 cells·mL^−1^ (2000 cells/well in a 96‐well plate). Cells were plated in a 50 μL volume so that, 24 h later, after addition of 50 μL of media with either the compounds or diluted DMSO, the final volume reached 100 μL. Cell viability was evaluated 48 and 72 h after the treatment. To that purpose, 20 μL of the MTS tetrazolium reagent was added to each well, and after 2 h of incubation at 37 °C, absorbance was measured at 490 nm.

### 2.5 Migration assays

For wound healing migration assays, G144 and G179 cells were seeded in Ibidi 2‐well Culture‐Inserts (Ibidi GmbH, Gräfelfing, Germany) at a cell density of 35 000 cells·cm^−2^. Twenty‐four hours later the insert was removed, and medium was replaced with the corresponding treatment. The wound closure was evaluated using phase‐contrast images (acquired with a phase‐contrast microscope Leica DMI3000) and calculated as the percentage of the area covered by cells after 24 h in comparison with the initial cell‐free area of each wound. Analysis was performed using the imagej software [54] and the MRI Wound Healing Tool.

For the three‐dimensional (3D) invasion assays, G144 cell spheroids were first formed by seeding G144 cells (40 000 cells·mL^−1^) in the absence of laminin for 8 days. Then, they were embedded in a 2 : 1 mix of MATRIGEL® (Corning, New York, NY, USA) and culture media. Matrigel mix was let to polymerize at 37 °C for 30 min, and it was subsequently covered with 100 μL of media containing the tested treatment. Each sphere was tracked individually over time, and the ratio between the invaded area 24 and 48 h after treatment versus the spheroid initial area was calculated. Analysis was performed using the imagej software.

### 2.6 Immunofluorescence and confocal image acquisition

Cells seeded on coverslips were fixed with 3.7% paraformaldehyde for 15 min at room temperature (RT) and immunostaining was performed as described previously [55]. Corresponding primary and secondary antibodies are detailed in Table S1. Confocal microscopy images (8 bits) were obtained in a Zeiss LSM800 Confocal Laser Scanning Microscope using 63 × Plan‐Apo/1.4 numerical aperture oil objective at RT. In some cases, a 0.5 zoom was used. Confocal Z‐stack images were acquired in all cases and stacks were z‐projected to maximum intensity. Images were processed with the zen blue software (Carl Zeiss Microscopy GmbH, Oberkochen, Germany). When necessary, image quantification was performed using the imagej software.

### 2.7 RNA isolation, reverse transcription, and quantitative real‐time PCR (qRT‐PCR)

Total RNA isolation and cDNA synthesis were performed using the RNeasy Mini Kit (Ref: 74106; Qiagen) and the High‐Capacity RNA‐to‐cDNA™ Kit (Applied Biosystems, Foster City, CA, USA), respectively. Gene expression levels were detected by qRT‐PCR in a StepOnePlus™ Real‐Time PCR System (Applied Biosystems) using FastStart Universal SYBR Green Master mix (Roche, Basel, Switzerland). All protocols were performed according to the manufacturer's instructions. Primer sequences are listed in Table S2.

### 2.8 Flow cytometry analysis

Quantification of CD133 levels and apoptosis percentages was performed in a MACSQuantify Analyzer (Miltenyi Biotec, Bergisch Gladbach, Germany) using macsquantify software. Detection of the CD133 marker was carried out with an APC‐conjugated CD133 antibody (AC133 clone; Miltenyi Biotec) or the corresponding IgG control at a 1 : 50 dilution. Up to 1 × 10^7^ cells were stained for 10 min at RT and analyzed immediately after staining. Apoptosis was assessed using the FITC Annexin V Apoptosis Detection Kit I (Ref: 556547; Becton Dickinson, Franklin Lakes, NJ, USA) following the manufacturer's instructions.

Cell cycle was analyzed using a CytoFLEX S (Beckman Coulter) cytometer. G144 cells treated for 72 h with BMT9 (2.25 μm) or the corresponding DMSO volume (0.1 μL·mL^−1^) were fixated in 70% ethanol at −20 °C overnight. Cells were stained with a solution of 1× PBS, 1 mm Na Citrate, 20 μg·mL^−1^ propidium iodide, and 20 μg·mL^−1^ RNAse A. flowjo™ v10.8 Software (BD Life Sciences, Franklin Lakes, NJ, USA) was used for data analysis, applying the Watson Pragmatic algorithm without constraints.

### 2.9 Western blot analysis

Protein isolation and electrophoresis were performed as published previously [33]. Membranes were incubated with the corresponding primary antibody (Table S1) with gentle agitation. After washing with Tris Buffer Saline‐0.05% Tween 20 (TBS‐T), membranes were incubated with 25 ng·mL^−1^ horseradish peroxidase‐coupled secondary antibodies (Pierce, Appleton, WI, USA) for 1 h at RT. HRP‐conjugated proteins were visualized with Super Signal West‐Pico Chemiluminescent Substrate (Thermo Fisher Scientific, Waltham, MA, USA), followed by membrane autoradiography or by image scan using a LI‐COR Odyssey Imaging System (LI‐COR Biosciences, Lincoln, NE, USA). Densitometry analysis was performed using the imagej software.

To precisely identify the different p73 isoforms, protein controls for TAp73α, TAp73β, and DNp73β were obtained. For this purpose, E14TG2a mouse embryonic stem cells (CVCL_9108) were transfected as described previously [56] with 1 μg of the following plasmid vectors: pcDNA3 CMV‐HA‐TAp73α, pcDNA3 CMV‐HA‐TAp73β, or pcDNA3 CMV‐HA‐DNp73α [57]. Protein samples were obtained 24 h after transfection.

### 2.10 RNA sequencing and transcriptome data analysis

For RNA sequencing (RNA‐seq) analysis, G144 cells were seeded at 4500 cells·cm^−2^ and 24 h later, the cells were treated with BMT9 (4.5 μm) or the corresponding volume of DMSO (0.2 μL DMSO·mL^−1^) for 48 h. Total RNA from three biological replicates for each condition was isolated using the RNeasy mini kit (Qiagen). G144 p73KO2 cells were seeded under the same conditions.

RNA sequencing was carried out by Novogene (Beijing, China) using the Illumina Sequencing PE150 system, and 40 million reads per direction were generated for each library. Mapping of the reads to the reference genome, normalization to obtain the FPKMs (fragments per kilobase of transcript per million mapped reads), and DESeq2 differential expression analysis were performed at the core service Bioinformatica USAL (Universidad de Salamanca, Spain). Quality of sequencing was validated with fastqc software [58]. The alignment and Quantification of the reads on the reference sequences was carried out with the salmon software [59]. The differential expression analysis was performed using the R statistical analysis program, with the deseq2 package [60]. Genes with an adjusted *P*‐value (*P*‐adj) < 0.01 were assigned as differentially expressed (DEGs).

Gene Ontology (GO) analysis was carried out using DAVID Bioinformatics Resources 6.834 [61, 62] choosing level 3 of GO terms specificity. For Gene Set Enrichment Analysis (GSEA), we used the software available at the Broad Institute website (https://www.gsea‐msigdb.org/gsea/index.jsp). Analysis was conducted on a gene list that was preranked by the log_2_ fold‐change values from the deseq2 analysis. The following Molecular Signatures Databases (MsigDB) were examined: ‘H: Hallmark Gene sets’, ‘C2: curated gene sets’, ‘C5: ontology gene sets’, and ‘C8: cell type signature gene sets’ [63, 64]. The statistical significance of the enrichment score (ES) was estimated by running 1000 gene set permutations, applying a weighted scoring scheme.

### 2.11 Statistical analysis

Statistical analysis and graphical data representations were generated using graphpad prism 9.5 software (GraphPad Software, Boston, MA, USA). Data are expressed as mean ± standard error of the mean (SEM). To test for normal distribution, Kolmogorov–Smirnov test was performed. Student's *t*‐test, one‐way ANOVA, or two‐way ANOVA were used to test single or multiple comparisons. As indicated in the figure legends, Tukey's or Dunnett's multiple comparisons tests were applied. When data followed a non‐normal distribution, the non‐parametric counterparts of each test were applied. Differences were considered significant when *P*‐value < 0.05.

## 3 Results and Discussion

### 3.1 *TP73* function is required for preserving glioblastoma stem cell biology

**Fig. 1:**
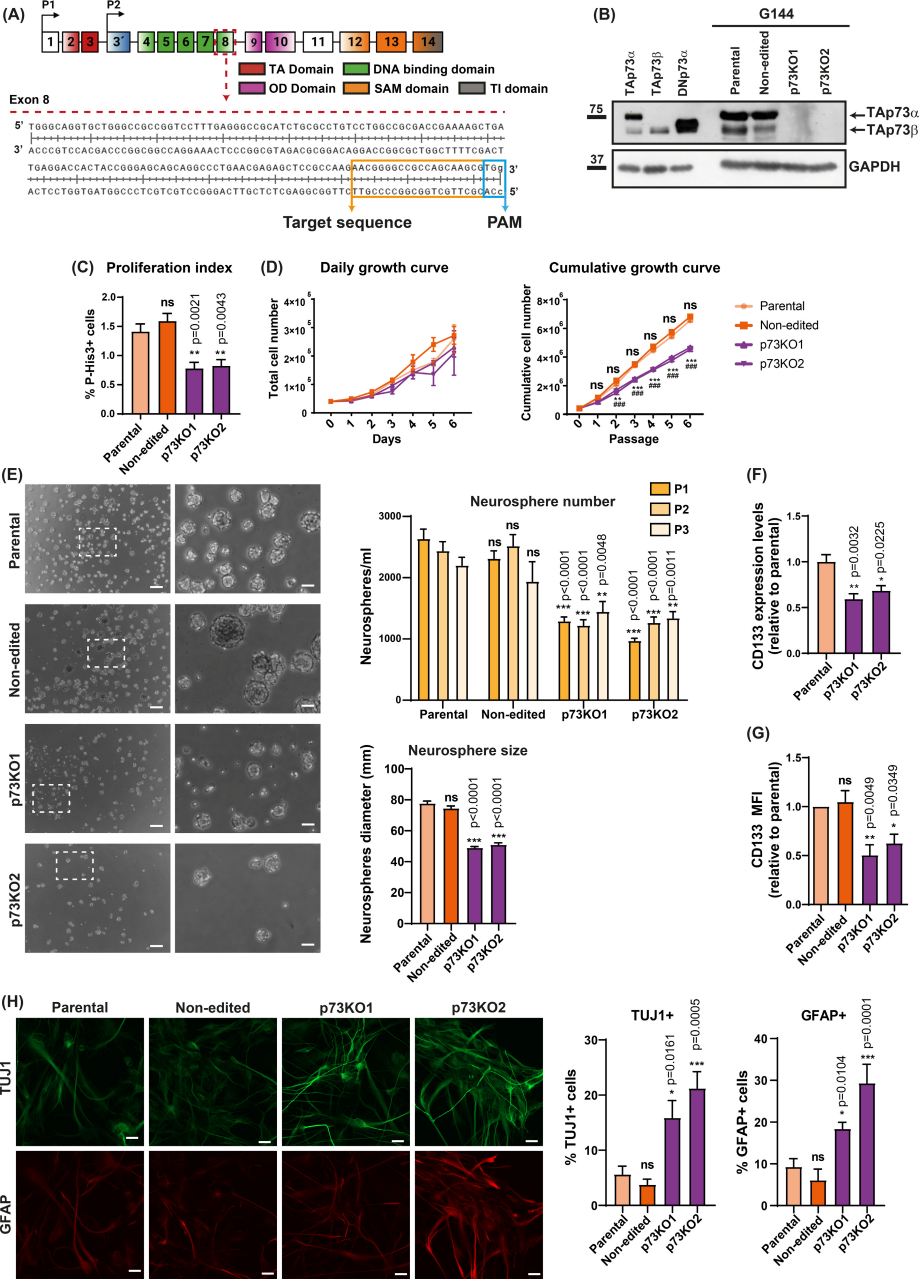
Generation and characterization of G144 *TP73* knockout (p73KO) glioblastoma stem cell lines. (A) Graphical representation of the *TP73* gene, indicating the single‐guide RNA (sgRNA) target sequence (orange square frame) located at Exon 8. OD, oligomerization domain; PAM, protospacer adjacent motif; SAM, sterile alpha‐motif domain; TA, transactivation domain; TI, transactivation inhibitory domain. (B) Western blot analysis of the selected G144‐edited clones using a pan‐p73 antibody (*n* = 3). GAPDH was used as the loading control. (C) Percentage of phospho‐Histone3 positive cells (p‐His3+) relative to the total number of cells (evaluated by DAPI staining) (*n* = 4). One‐way ANOVA with Tukey's multiple comparisons test was used to address statistical differences versus parental cells; ns, non‐significant. (D) Growth kinetics analysis of the G144‐p73KO clones. Left graph: daily growth curves of the indicated genotypes (*n* = 3). Two‐way ANOVA revealed no statistical differences. Right graph: cumulative growth curves generated by splitting the cells every 4 days to ensure maintenance in the exponential growth phase (*n* = 3). Two‐way ANOVA with Dunnett's multiple test was used to compare p73KO1 cells (*), p73KO2 cells (#), and non‐edited cells with parental cells. (E) Neurosphere (NS) formation analysis. Left panel: representative phase‐contrast images of 8‐days NS plated at clonal density conditions (scale bar: 250 μm). Magnifications of the areas marked with white dashed squares are shown (scale bar: 60 μm). Right panel top graph: quantification of the number of NS per mL at the indicated consecutive passages (*n* = 3). Statistical differences were estimated with respect to the parental cells at each passage (P1, P2, and P3) using two‐way ANOVA with Dunnett's multiple comparisons test. Right panel bottom graph: average NS size (diameter) of at least 30 NS per condition (*n* = 3). Kruskal–Wallis test with multiple comparisons (Dunn's test) was performed to analyze statistical differences versus the parental cells. (F) qRT‐PCR analysis of CD133+ expression levels (*n* = 3). One‐way ANOVA with Tukey's multiple test was used to estimate statistical differences (comparison with the parental cells). (G) Flow cytometry analysis of CD133 protein expression (*n* = 5). The mean fluorescence intensity (MFI) of CD133 was normalized to the corresponding IgG isotype control; then, relative values to the fluorescence of parental cells were calculated and plotted. Statistical differences with respect to the parental cells were determined by one‐way ANOVA followed by Tukey's multiple test. (H) Analysis of the differentiation state of p73KO clones under self‐renewal conditions by immunofluorescence and confocal microscopy. Left panel: representative confocal micrographs of cells double‐stained for Tuj1 (green) and GFAP (red). Scale bar: 20 μm. Right panel: quantification of the percentage of Tuj1+ and GFAP+ cells. At least 100 cells from three independent experiments were analyzed per condition. One‐way ANOVA with Tukey's multiple comparison test was used to address statistical differences (comparison with the parental cells). All the graphs display the mean value ± SEM (standard error of the mean); ns, non‐significant; *P < 0.05, **P < 0.01, *** or ^###^ *P* < 0.001.

The growth‐promoting role of *TP73* in brain tumors and, in particular, TAp73 requirement for CSCs maintenance has been previously postulated [12, 14]. Thus, we sought to explore the effect of *TP73* inactivation on GSC stemness. To this purpose, we chose the G144 cell line as a model. This adherent cell line, derived from an *IDH1* wild‐type human malignant glioma, displays stem cell properties and maintains self‐renewal and multipotency when cultured under 2D conditions [44]. We generated *TP73* knockout (p73KO) derivatives of G144 cells via CRISPR/Cas9 gene editing, targeting Exon 8, which is common to all p73 isoforms (Fig. 1A). We obtained two homozygous edited clones, named G144‐KO1 (p73KO1) and G144‐KO2 (p73KO2), in which sequencing confirmed a single nucleotide frame‐shifting insertion of an adenine (Fig. S1A) that resulted in a truncated protein with a completely altered sequence encoding the oligomerization domain [15] and lacking the C terminus domain (Fig. S1B). Additionally, another clone that was subjected to the same gene editing procedure, but with no change in sequence, was selected as a non‐edited control. qRT‐PCR analysis using primers that match the target sequence confirmed *TP73* gene inactivation in both clones (Fig. S1C). Analysis of gene expression in G144 parental and non‐edited cells using a pan‐p73 antibody showed a strong band with similar mobility to TAp73α (Fig. 1B), which was confirmed by western blot analysis with a TA‐specific antibody (Fig. S1D). We detected very low levels of DNp73 by qRT‐PCR (Ct = 34.5 versus Ct = 28.6 for TAp73) and no detectable protein with electrophoretic mobility corresponding to DNp73 (Fig. 1B), all indicating that TAp73 is the predominant isoform and, thus, the one responsible for p73 activity in these cells. We did not detect any p73 protein bands in the p73KO1 and p73KO2 clones (Fig. 1B and Fig. S1D), indicating that we have successfully inactivated *TP73* in G144 GSCs.

The edited G144 lines could grow, like their parental and non‐edited counterparts, under 2D adherent culture conditions forming monolayers (Fig. S1E). G144 cells have been described to have similarities to fetal NSCs, containing two types of morphologies with dynamic interconversions: small polar cells (Polar cells) and more flattened apolar cells with short extensions (Apolar cells) [43, 44]. When seeded under low‐density conditions, the polar cells are the predominant in the parental G144 cultures, which, like fetal NSCs, have elongated bipolar morphology, lamellate extensions, end‐feet, and oval nuclei reminiscent of radial glia cells [44, 50] (Fig. S1F and Video S1). However, the p73‐deficient cells preferentially adopted the flattened apolar shape (Fig. S1F and Video S2), suggesting that the lack of p73 could be affecting the NSC‐like features of this GSC line.

Analysis of the mitotic rate by phospho‐Histone 3 (P‐His3) staining revealed that G144 parental cells have a low proliferation index (around 1.5% of P‐His3+ cells); nevertheless, p73 deficiency resulted in a significant reduction of this rate (Fig. 1C). This difference was not reflected in short‐term growth curves (Fig. 1D, left panel) but was clearly exposed in cumulative growth curves, where p73KO cells showed a significant decrease in the cumulative cell number with time (Fig. 1D), suggesting that p73 inactivation impairs the long‐term auto‐replicative capacity of these cells.

The ability to self‐renew is one of the properties employed by the CSCs to maintain their stemness potential and tumor‐initiating faculty [65]. It has been previously demonstrated that lack of p73 impairs self‐renewal in NSCs [42]. To address whether p73 inactivation would similarly hinder GSC self‐renewal, we analyzed the number of neurospheres (NS) formed under clonal culture conditions in successive passages. The control cells, including the non‐edited, formed significantly more NS, and with higher diameter (Fig. 1E), than p73KO cells, which were unable to form NS efficiently in subsequent passages, demonstrating that the growth and self‐renewal capacity of G144 GSCs gets impaired in the absence of p73.

Self‐renewal implies cell division while preserving the undifferentiated state [66]. Therefore, we analyzed whether the observed defect on self‐renewal also resulted in an increase in spontaneous differentiation under self‐renewing culture conditions. Flow cytometry analysis revealed a strong and significant decrease in the expression of the GSCs marker CD133, at RNA and protein levels (Fig. 1F,G, respectively), in the p73KO clones under proliferative conditions compared with controls. Furthermore, under these culture conditions and correlating with the decrease in CD133, p73KO clones showed a significant increase in the Tuj1+ and GFAP+ populations (Fig. 1H). On the contrary, control cells remained mostly undifferentiated, with < 10% of the cells undergoing differentiation (Fig. 1H), altogether suggesting that p73 deficiency may promote spontaneous multilineage differentiation in GSCs, similarly to its known effect on NSCs [41, 42]. These results confirmed that p73 plays a critical role in maintaining the core stem cell properties of GSCs, which encompass a set of molecular processes that have been referred to as ‘stemness’ [67]. As TAp73 is the major isoform in G144 cells, it is reasonable to postulate that the detected differences upon p73 inactivation in these cells are due to the elimination of TAp73 function.

**Fig. 2:**
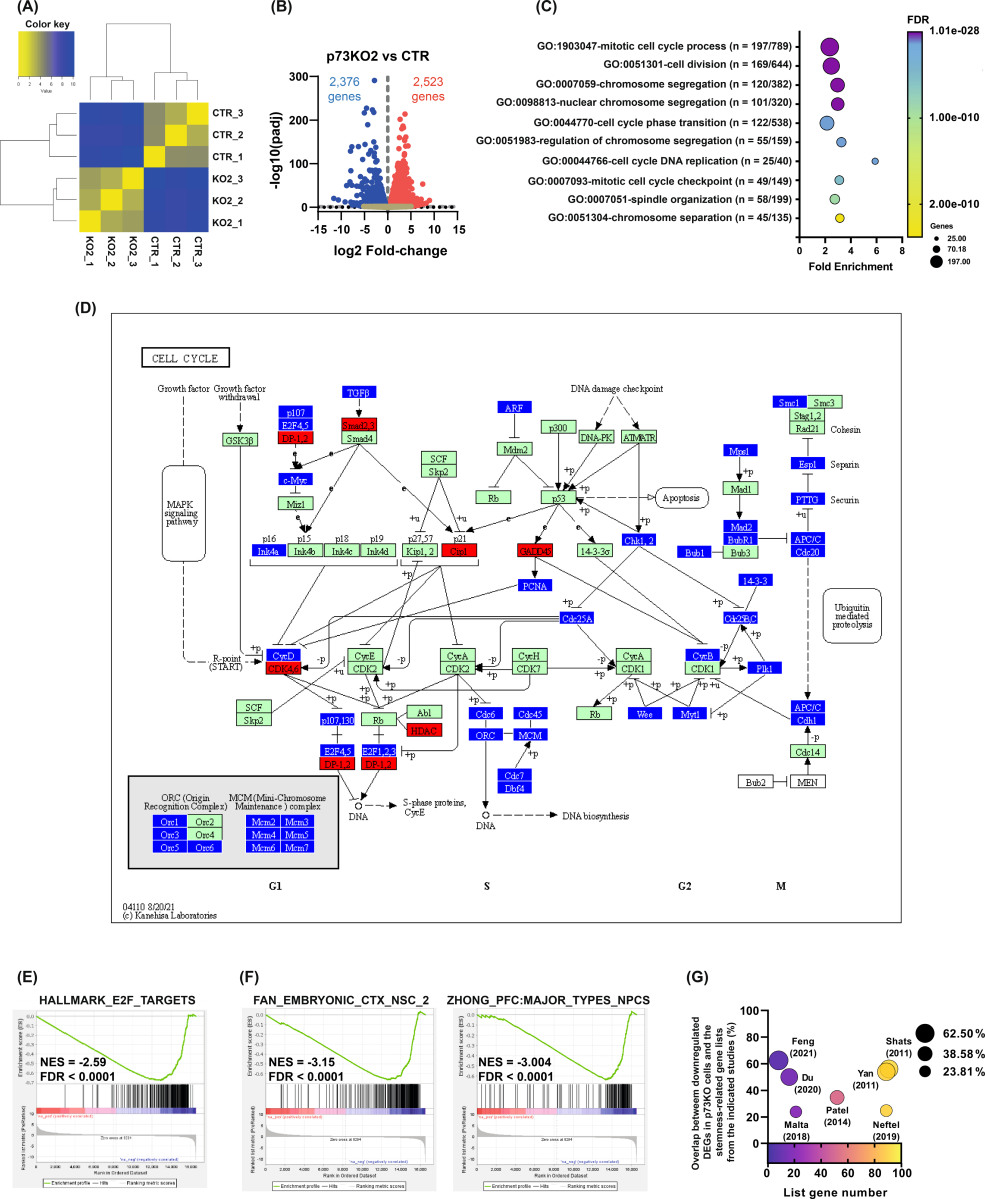
Transcriptomic analysis of *TP73* knockout (p73KO) cells compared with parental cells. RNA sequencing data were generated using three replicates per condition. (A) Hierarchical clustering analysis showing the correlation of gene expression for all pairwise combinations of the samples in the dataset (CTR: G144 parental cells; KO2: p73KO2 cells). (B) Volcano plot representing the magnitude of gene expression change (log_2_ fold change) of differentially expressed genes (DEGs) between the p73KO and the parental cells, plotted against the statistical significance (−log_10_ of the adjusted *P*‐value, *P*‐adj). Red and blue dots indicate the upregulated and downregulated DEGs, respectively. Genes that were not differentially expressed are displayed as light brown dots. (C) DAVID Functional annotation analysis of the downregulated DEGs (*P*‐adj < 0.01) using the Gene Ontology (GO) Biological Process dataset. The 10 most statistically significant terms with at least a twofold enrichment are displayed. The dot color indicates the false discovery rate (FDR), while the dot size illustrates the number of DEGs annotated to that term. (D) Chart of the ‘Cell cycle pathway’ (hsa04110) from the Kyoto Encyclopedia of Genes and Genomes (KEGG) highlighting the upregulated (red boxes) and downregulated DEGs (blue boxes) in p73KO cells. (E, F) Gene Set Enrichment Analysis (GSEA) performed on the whole DEG list (preranked by log_2_ fold‐change values) compared with the ‘H: Hallmark Gene sets’ (E) and the ‘C8: cell type signature gene sets’ (F). The normalized enrichment score (NES) and FDR are indicated for each plot. The graph on the bottom of each plot represents the ranked, ordered, non‐redundant list of genes. (G) Bubble plot representing the overlap between the downregulated DEGs in p73KO cells and the stemness‐related gene lists from the indicated studies. Horizontal axis and color gradient depict the number of genes included in the respective stemness lists. Vertical axis and bubble size indicate the percentage of overlap between each study and our downregulated DEG list.

To dissect the molecular pathways mediating p73 role in GSC stemness, we performed a global transcriptome analysis comparing G144 control cells (CTR) and p73KO cells by RNA‐seq (Table S3). Unsupervised hierarchical clustering identified two well‐defined transcriptional phenotypes corresponding to the parental and the p73KO samples (Fig. 2A). Highlighting the significant transcriptomic changes as a result of p73‐elimination, analysis of differentially expressed genes (DEGs) (Fig. 2B) revealed 2523 upregulated genes (from which 553 were robustly induced in the absence of p73, log_2_FC > 2.0) and 2376 downregulated genes (with 256 genes whose transcription was strongly repressed in p73KO cells, log_2_FC < −2.0). To identify transcriptional networks specifically activated by p73, we performed a functional annotation analysis focusing on the downregulated DEGs in p73KO cells. The most statistically significant GO terms with at least a twofold enrichment were related to cell division and mitotic cell‐cycle progression, chromosome segregation, spindle organization, and the mitotic assembly checkpoint (Fig. 2C). A similar analysis using the Reactome pathway database further supported these results, with the ‘Cell cycle pathway’ (R‐HSA~1640170) having the highest significance (FDR 1.32 × 10^−42^ cells; fold enrichment = 2.6; 228 genes included). To extract the most relevant genes for cell‐cycle progression affected by p73 elimination, we compared the whole set of DEGs with the KEGG database ‘Cell Cycle pathway’ (hsa04110) (Fig. 2D and Table S4). As depicted in the pathway map, p73 inactivation in these cells mostly resulted in downregulation of several relevant cell‐cycle regulation factors, including various members of the E2F1 family, required for correct cell‐cycle progression [68]. GSEA analysis on the whole DEGs list confirmed that p73 inactivation resulted in downregulation of E2F1 targets and E2F1 signaling signature (Fig. 2E) in GSCs. Furthermore, some of the most relevant cell‐cycle repressors and senescence regulators like p21/CDKN1A/Cip1 (*P*‐adj: 0.0029), GADD45B (*P*‐adj: 8.82 × 10^−5^), and GADD45G (*P*‐adj: 0.017) were upregulated in the p73KO cells. Altogether, these data indicated that TAp73 acts as a crucial transcription factor that sustains cell growth and proliferation in GSCs.

To evaluate the extent of association between the observed transcriptomic changes and p73 regulation of stemness, we performed a GSEA against different cell type signature gene sets (MSigDB‐C8). In agreement with p73 known role as a regulator of NSC and supporting its involvement in the maintenance of GSC stemness, GSEA analysis revealed that gene signatures related to NSCs [69] and neural progenitor cells [70] were negatively affected by p73 loss (Fig. 2F), suggesting that p73 could regulate stemness maintenance through mechanisms shared by GSCs and NSCs. Multiple studies have tried to identify a stemness‐associated transcriptional signature for GSCs, but a consensus gene list is still missing. Therefore, we revisited some of these studies [71, 72, 73, 74, 75, 76, 77] (Table S5) and compared the identified gene signatures with our list of downregulated DEGs. This comparison revealed that a high percentage of genes from the published stemness signatures were common to our downregulated DEGs in the p73KO GSCs (Fig. 2G). The higher number of overlapping genes was obtained when comparing our list with the gene signatures from Shats et al. [76] (51 genes from 91) and Yan et al. [77] (48 from 89), which mostly represent the transcriptome profile associated with CD133+ GSCS. Taken together, our results indicate that p73 regulates gene networks related to GSCs growth and stemness, and that it is critical to maintain the stemness potential in these cells. As mentioned above, TAp73 is the major p73 protein isoform in G144 cells; therefore, it is reasonable to conclude that the detected differences upon *TP73* inactivation in these cells are due to elimination of TAp73 function.

### 3.2 Screening of a small natural compound library reveals a novel candidate drug with TAp73 inhibitory activity

It has been widely proposed that the repression of stemness‐associated signaling pathways could ultimately improve the treatment of cancer [78, 79]. Furthermore, a recent report suggests that in tumors lacking wild‐type p53, ablation of E2F family members—as well as p73—causes cell‐cycle arrest, indicating that, in the absence of p53, p73 regulates cell‐cycle progression, making it a potential target of interest in these cancers [80]. Therefore, we and others hypothesized that downregulation of TAp73 could be an interesting therapeutic strategy in GB [14, 81].

**Fig. 3:**
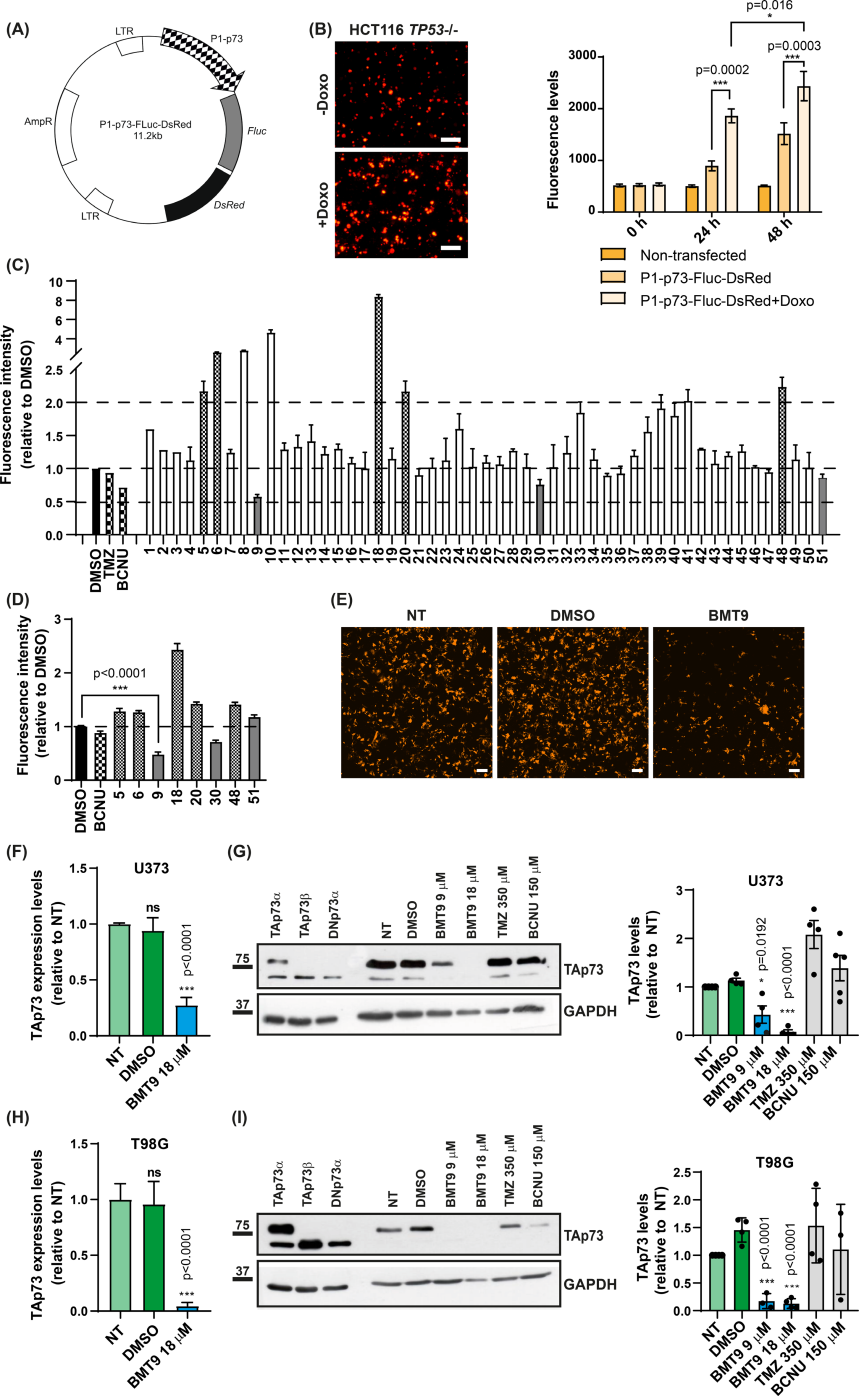
Identification of a novel natural compound with TAp73 repressing capacity. (A) Map of the LV‐P1‐*TP73*‐Fluc‐DsRed2 reporter vector. (B) Validation of the reporter system in HCT116 *TP53*−/− cells. Left panel: representative images of cells transfected with the reporter vector and treated with of Doxorubicin (± Doxo, 1 μm). Scale bar: 200 μm. Right panel: quantification of fluorescence levels by fluorimetry (*n* = 3). Statistical differences were calculated using a two‐way ANOVA with Tukey's multiple comparisons test. (C) Fluorimetry analysis from the screening of 51 natural compounds from Biomar Microbial Technologies performed in U373 cells (8 μg·mL^−1^ of the tested compounds; DMSO: 0.8 μL·mL^−1^ media (*n* = 1). Treatments with Temozolomide (TMZ) and Carmustine (BCNU) were also included. (D) Validation of compounds showing any detectable repressing activity in C) (gray bars) or a modulatory effect ≥ 2‐fold (square pattern) by fluorimetry (*n* = 3). Mann–Whitney test was used to compare fluorescence intensity between the compounds and DMSO‐treated cells. (E) Fluorescence microscopy of cells treated with the selected compound, BMT9. Representative images from three independent experiments are shown. NT corresponds to non‐treated cells. Scale bar: 200 μm. (F–I) Confirmation of TAp73 repressing activity by BMT9 in U373 (F, G) and T98G (H, I) conventional glioblastoma cell lines by qRT‐PCR (F and H, *n* = 3) and western blot (G and I, *n* = 4). In F and H, one‐way ANOVA with Dunnett's multiple test was used to analyze statistical significance. For G and I, unpaired *t*‐tests were performed. In all cases (F–I), comparisons with the non‐treated cells are shown. All the graphs display the mean value ± SEM (standard error of the mean); ns, non‐significant; *P < 0.05, ***P < 0.001.

To that purpose, we engineered a bimodal reporter vector (LV‐P1‐*TP73*‐Fluc‐DsRed2), based on a published lentiviral system [52], where we swapped the original CMV promoter for a 930‐bp fragment of the human P1‐*TP73* promoter [53] (Fig. 3A). The basal activity of P1‐*TP73* promoter was easily detected by fluorescent microscopy 48 h after the vector transfection in different human cell lines (Fig. S2A). To validate the generated system, we transfected the reporter construct in HCT116 p53−/− cells, in which endogenous TAp73 can be induced by the genotoxic drug doxorubicin (Doxo) [82] at RNA and protein levels (Fig. S2B,C). A significant increase in fluorescence was detected after Doxo treatment of transfected cells at the analyzed time points (Fig. 3B), indicating that the engineered reporter could detect drug‐induced changes in the P1‐*TP73* promoter that correlated to endogenous TAp73 expression.

To identify repressors of TAp73 expression, we performed a low‐throughput chemical screen of a panel of 51 natural compounds (BMT1 to BMT51) with novel chemical structures and previously selected by Biomar Microbial Technologies for their effect against GB cells but with low to moderate cytotoxicity. Transfected U373 GB cells were treated with either 8 μg·mL^−1^ of the tested compounds, the drugs used as first‐line treatments (TMZ and BCNU) or the equivalent volume of DMSO. The reporter activity was quantified 48 h later and normalized to the control DMSO‐exposed cells (Fig. 3C). Compounds with autofluorescence, like BMT8 and 10, were discarded whereas the compounds with either any detectable repressing activity or with a modulatory effect ≥ 2‐fold were preselected (BMT5, BTM6, BTM9, BTM18, BTM20, BTM30, BTM48, and BTM51). Using the same experimental design and methodology, we validated the reporter modulatory activity of the preselected compounds to obtain significant statistical values (Fig. 3D). The only compound with a significant and consistent transcriptional repression of P1‐*TP73* promoter activity was BMT9 (Fig. 3D,E), and therefore, it was selected for further study. TAp73 expression analysis at the screening concentration demonstrated that BMT9, but not TMZ or BCNU, significantly downregulated endogenous TAp73 at RNA and protein levels in conventional GB cell lines: U373 (Fig. 3F,G) and T98G (Fig. 3H,I). Altogether, we have identified a new natural compound with TAp73‐inhibitory capacity in GB cells. This compound corresponds to a new meroterpene, a natural product of mixed polyketide/terpenoid biosynthesis isolated from a fungal strain from Biomar Microbial Technologies (patent number EP22382943.3).

### 3.3 The novel compound BMT9 is more effective against glioma stem cells than first‐line glioblastoma treatments TMZ and BCNU

**Fig. 4:**
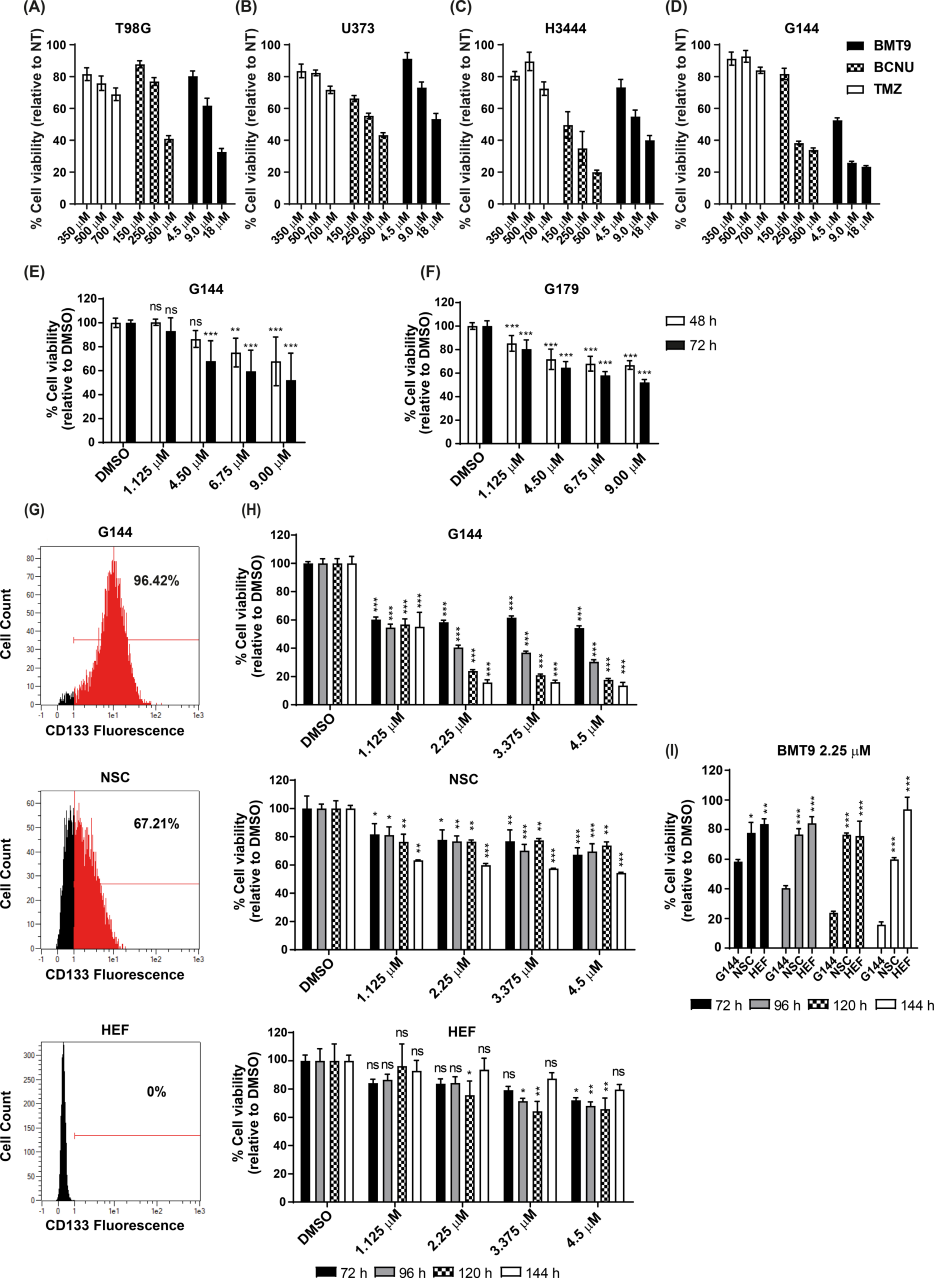
Comparative analysis of BMT9 on cellular viability. (A–D) Dose‐dependent effect of BMT9 compared with TMZ and BCNU in conventional glioblastoma cell lines (T98G, U373, and H3444) (A–C) and the stem‐like glioblastoma cells (GSCs) G144 (D) (*n* = 3). Cells were treated with the indicated drug concentrations for 72 h. Cell viability was determined by MTT‐viability assay, and values were normalized to the non‐treated (NT) cells. (E, F) Cell viability analysis of GSCs G144 (E) and G179 (F) treated with BMT9 for 48 and 72 h (*n* = 3). Values were normalized to the DMSO‐treated cells (0.4 μL·mL^−1^ media). Statistical differences between each treatment and the respective control cells were estimated for each time point using a two‐way ANOVA and Dunnett's multiple comparison test. The graphs display the mean value ± SEM (standard error of the mean); ns: non‐significant; **P < 0.01, ***P < 0.001. (G) Quantification of CD133‐positive cells (red area in the histogram) by flow cytometry analysis of G144 cells (upper panel), a healthy human fetal neural stem cell line (NSC) (middle panel), and a human embryonic fibroblast (HEF) cell line (bottom panel). The negative population was gated using the appropriate IgG isotype control (black area). (H) Viability assessment after BMT9 treatment in long‐term cultures of G144, NSC, and HEF (*n* = 3). Cells were treated with the indicated doses of BMT9 and MTT‐viability assays were performed up to 144 h. Values were normalized to DMSO control (0.2 μL·mL^−1^ media). Statistical differences between BMT9‐treated cells and the control were estimated for each time point and drug concentration using a two‐way ANOVA with Dunnett's multiple comparisons test. To facilitate the comparison between the three cell lines, values for the 2.5 μm concentration from (H) are graphically regrouped in (I), and the statistical differences versus the G144 cells are indicated at each time point (one‐way ANOVA with Tukey's multiple comparisons test). All the graphs display the mean value ± SEM; ns, non‐significant; *P < 0.05, **P < 0.01, ***P < 0.001.

To address whether BMT9 preferentially affected GSCs, the compound was tested against GB conventional cell lines T98G, U373, and H3444 [48], as well as the GSC lines G144 and G179 [44] (Fig. 4A–F). We performed a dose–response analysis comparing BMT9 (4.5–18 μm), with BCNU (150–500 μm) and TMZ (350–700 μm) using the MTT‐viability assay. The conventional cell lines were sensitive to BTM9 with an IC50 at 72 h between 9 and 18 μm (Fig. 4A–C). Interestingly, in G144 and G179, the IC50 at 72 h was even lower, between 4.5 and 9 μm, indicating that, while BMT9 was highly effective to all GB cell lines tested, GSCs showed higher sensitivity to it (Fig. 4E,F). On the contrary, the IC50s for the current drugs were more than 10‐fold higher: between 150 and 500 μm for BCNU, and over 700 μm for TMZ (in this case, we could not reach the IC50 in any of the cell lines for the tested doses; Fig. 4A–D).

Previous studies have reported that the molecular signature of the G144 cell line corresponds to a ‘proneural’ subtype of GB with NSCs features [44, 83]. Thus, to test BMT9 specificity, we compared the cellular response to this drug in CD133+ cell lines, such as G144 and FT430 (a healthy human fetal NSC line), versus human embryonic fibroblasts (HEF) which do not express CD133 (Fig. 4G). As with previous experiments, the IC50 was reached in G144 cells treated with 4.5 μm BMT9 for 72 h, but not in the other cell lines (Fig. 4H). Even though at this time point the viability of the NSCs was affected down to a 67.32% viability, this viability was maintained in time and the IC50 was never reached. Interestingly, when analyzing the long‐term effect (up to 144 h) using lower BMT9 concentrations (1.125–3.375 μm), G144 cells were quite affected by the 2.25 μm treatment, whereas NCSs were marginally affected, with a 76.50% survival after 120 h of treatment, compared with a 23.90% of viable G144 cells at that time point (Fig. 4I). Statistical analysis showed a significant difference in sensibility to BMT9 between G144 and healthy NSCs at this dose (2.25 μm) (Fig. 4I). In addition, the HEFs were quite resistant, with a cell viability of 79.63% at the higher dose used, and 93.70% at 2.25 μm in long‐term analysis, altogether indicating that BMT9 has specificity against CD133+ cells, with a stronger effect against GSCs, suggesting a therapeutic window for this compound.

### 3.4 BMT9 reduces TAp73 levels in GSCs and impairs their cell growth, migration, and invasiveness capacity

**Fig. 5:**
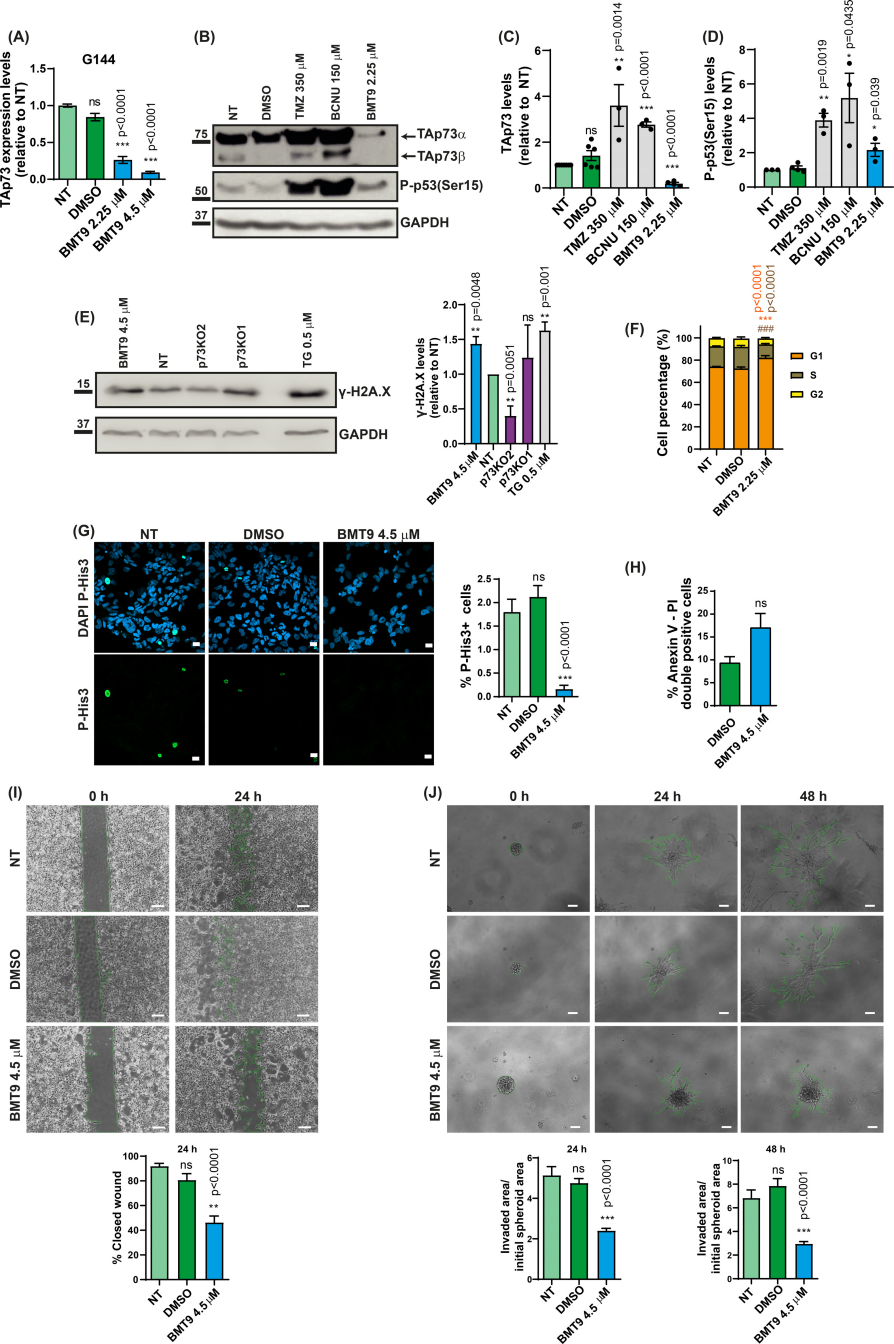
Characterization of the effect of BMT9 on stem‐like glioblastoma cells. (A) Analysis of TAp73 expression by qRT‐PCR in G144 cells treated with BMT9 for 72 h (*n* = 3). DMSO: 0.2 μL·mL^−1^ media. Expression data were normalized against non‐treated (NT) cells. Statistical differences (versus NT cells) were determined by one‐way ANOVA followed by Dunnett's multiple comparisons test. (B–D) Analysis of TAp73 (*n* = 7) and phosphorylated p53 (Serine 15) (*n* = 4) protein levels by western blot analysis after 72 h of treatment at the indicated doses. Protein quantifications were normalized to their respective loading control (GAPDH), and relative values to the NT cells are represented (C, D). Unpaired *t*‐tests were performed to determine the statistical significance (comparison with NT cells). (E) Analysis by western blot of γ‐H2A.X induction in BMT9‐treated cells, NT parental cells, or in p73KO cells (*n* = 3). Thapsigargin (TG) was used as a positive control of DNA damage induction. Relative γ‐H2A.X expression values to the NT parental cells are plotted (right graph), using GAPDH as a loading control. Unpaired *t*‐test was used to address statistical differences (comparison with NT cells). (F) Flow cytometry‐propidium iodide cell‐cycle analysis of NT, DMSO control (0.1 μL·mL^−1^ media), or BMT9‐treated G144 cells after 72 h (*n* = 3). Two‐way ANOVA and Tukey's multiple test were used to compare the G1 and S population percentage between BMT9‐treated cells and NT cells. *P*‐values are depicted in orange for G1 percentage comparison, and brown font for S percentage. (G) Proliferation index of G144 cells treated with BMT9 for 72 h, immunostained with phospho‐Histone3 (P‐His3), and analyzed by confocal microscopy (*n* = 3). Nuclei were counterstained with DAPI. DMSO: 0.2 μL·mL^−1^ media. Scale bar: 10 μm. Quantification of the percentage of pHis3+ cells relative to the total number of cells (right graph). At least 500 cells were analyzed per experiment. Significance was estimated by one‐way ANOVA and Tukey's multiple test (comparison with NT cells). (H) Apoptosis induction assessment by Anexin V‐FITC and propidium iodide (PI) staining and flow cytometry analysis in G144 cells after BMT9 treatment for 48 h. The average percentage of double‐positive cells from three independent experiments is shown. DMSO: 0.4 μL·mL^−1^ media. Unpaired *t*‐test was used to calculate statistical significance (comparison with DMSO‐treated cells). (I, J) Analysis of BMT9 effect on migration and invasion of G144 cells using the wound healing (I) (*n* = 4) and 3D invasion assay (J) (*n* = 3), respectively. DMSO: 0.2 μL·mL^−1^ media. Quantified areas are marked in green. Scale bar: 250 μm (I) and 100 μm (J). In both cases, one‐way ANOVA with Dunnett's multiple comparisons test was used to analyze statistical differences (BMT9‐treated cells compared with NT control cells). All the graphs display the mean value ± SEM (standard error of the mean); ns: non‐significant; *P < 0.05, **P < 0.01, ***P < 0.001.

We have demonstrated that BMT9 repressed TAp73 expression in conventional GB cell lines. Thus, we asked whether the TAp73‐inhibitory effect was also effective in G144 and G179 GSCs. BMT9 treatment strongly downregulated TAp73 RNA levels in both cell lines (Fig. 5A and Fig. S3A, respectively). For protein analysis, and to avoid excessive cell death in the analyzed cultures, we used a sub‐IC50 dose of BMT9 (2.25 μm for 72 h). As expected for genotoxic agents, TMZ and BCNU induced the expression of TAp73 proteins in G144 and G179 (Fig. 5B,C and Fig. S3B,C, respectively). However, in line with the RNA results, BMT9 treatment drastically reduced TAp73 protein levels in both GSC lines, with the effect being more pronounced in G144 which are more sensitive to BMT9, suggesting that the compound mode of action could be, at least partially, linked to its inhibitory effect on TAp73.

Additionally, we examined p53 activation by detecting the phosphorylation state of Serine 15 [P‐p53(Ser15)] in G144 and G179 GSCs (Fig. 5B,D and Fig. S3B,D, respectively). In both cell lines, TMZ and BCNU strongly upregulated P‐p53(Ser15) levels (G144: 3.89‐fold TMZ, 5.18‐fold BCNU; G179: 13.63‐fold TMZ, 15.24‐fold BCNU, in this case *P*‐value = 0.06). BMT9 only induced a moderated 2.16‐fold increase of P‐p53(Ser15) in G144 cells (Fig. 5B,D); however, it could trigger a DNA damage response in a p53‐independent manner. Moreover, it has been previously reported that p73 deficiency could be associated with increased DNA damage in certain cellular contexts [84]. Thus, we analyzed whether BMT9 treatment, or elimination of p73 in the knockout clones, resulted in γ‐H2AX induction in these cells. We used Thapsigargin (TG) as a positive control, since it can induce a mild DNA damage response [85] in a p53‐independent manner [86]. Our data indicate that p73 elimination alone is not capable of triggering a significant DNA damage response in these cells whereas BMT9 induces a small but significant increase in γ‐H2AX similar to that of TG (Fig. 5E), reflecting a weak DNA damage response. Altogether, this hints toward additional mechanisms of action beyond the genotoxic stress response signaling pathway.

Since our data indicated that TAp73 acts as a crucial transcription factor that sustains cell growth and proliferation in GSCs, we next analyzed the effect of BMT9 on cell proliferation. Cell‐cycle analysis (Fig. 5F) revealed that while untreated cells showed a high G1 population (74.2%), similar to cells treated with DMSO (72.4%; *P* = 0.1708), BMT9 caused an increase in this G1 population (up to 82%; *P* < 0.0001), together with a concomitant reduced proportion of cells in S phase (12% in BMT9‐treated cells versus 19.5% in DMSO control; *P* < 0.0001), indicating that BMT9 induces a G1‐phase cell‐cycle arrest at 2.25 μm concentration. Despite the low proliferation index of G144 cells, IC50 concentrations of BMT9 significantly decreased the percentage of cells in mitosis (Fig. 5G), while inducing apoptosis (Fig. 5H), even though not at significant levels (*P*‐value = 0.079), suggesting that the observed effect against GSCs at IC50 may not be due to massive induction of cell death. Nevertheless, at higher concentrations (9 μm), BMT9 can significantly induce apoptosis (68.69% of apoptotic cells). Remarkably, BMT9 affected one of the hallmarks of the GB cells, and of GSCs in particular, which is their invasive capacity [4, 87]. Using the wound healing assay in G144 and G179 (Fig. 5I and Fig. S3E, respectively) and a 3D spheroid cell‐invasion model (Fig. 5J and Fig. S3F, respectively), we demonstrated that BMT9 treatment significantly impaired the cell migration and invasiveness capacity of these GSCs *in vitro*. It is important to notice that, in this experiment, the compound was applied only for 24 h and, therefore, the described effect was observed at sublethal concentrations, since the reported IC50 is reached at 72 h. This suggests that the inhibitory effect of BMT9 on cell migration and invasion is not a consequence of a reduction in cell viability, but instead, it is a direct effect on these processes. Altogether, our results support an anti‐invasive role for BMT9 when used at sublethal concentrations in GSCs, reinforcing the interest in the BMT9 compound as a potential GB treatment.

### 3.5 BMT9 decreases GSC stemness and is effective against patient‐derived GSCs‐enriched lines

**Fig. 6:**
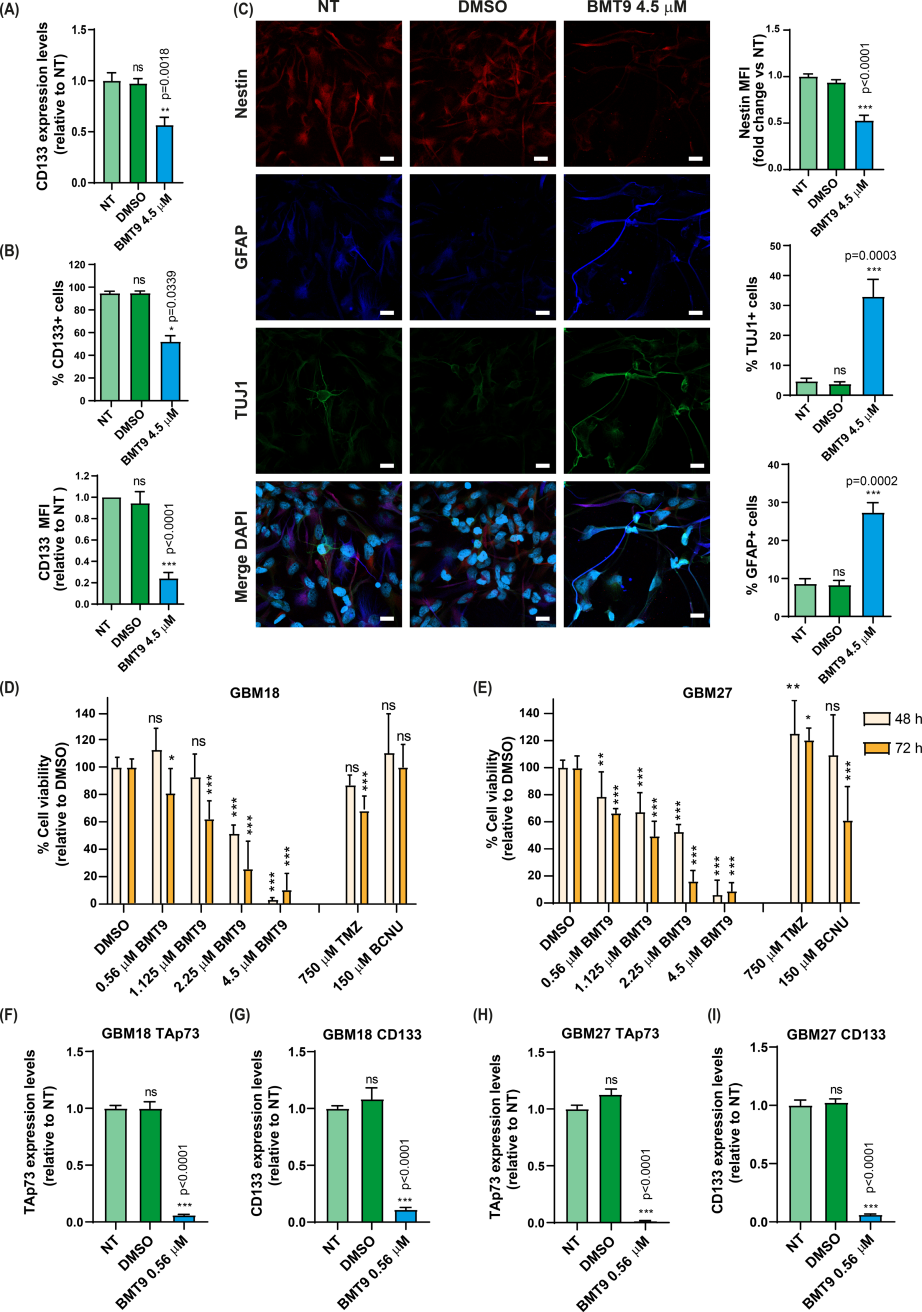
Effect of BMT9 on stemness of stem‐like glioblastoma cells (GSCs) and patient‐derived GSC enriched lines. (A) CD133 RNA expression levels in G144 GSCs after 48 h of treatment (DMSO: 0.2 μL·mL^−1^ media) (*n* = 3). One‐way ANOVA with Tukey's multiple comparison test was used to calculate statistical differences versus non‐treated cells (NT). (B) Analysis of the CD133+ population (percentage and mean fluorescence intensity – MFI) in G144 cells treated with 4.5 μm BMT9 for 48 h (*n* = 4). DMSO: 0.2 μL·mL^−1^ media. IgG isotype control was used to define the negative population and to normalize CD133 expression levels. Relative MFI values (to NT cells) are shown. In both cases, statistical differences versus NT cells were estimated using a one‐way ANOVA with Dunnett's multiple comparison test. (C) Analysis of differentiation induction by BMT9 on G144 cells under proliferating conditions (*n* = 3). Immunofluorescence and confocal microscopy of BMT9‐treated cells, DMSO (0.2 μL·mL^−1^ media) or the corresponding NT controls after 72 h treatment, using anti‐Nestin (red), anti‐GFAP (blue), or anti‐Tuj1 (green) antibodies. Nuclei were counterstained with DAPI. Scale bar: 20 μm. Graphs on the right correspond to the quantification of the Nestin mean fluorescence intensity (MFI) and the percentage of Tuj1+ and GFAP+ cells, as indicated. A minimum of 350 cells per condition were analyzed. Statistical differences in Nestin MFI were calculated using a one‐way ANOVA. For Tuj1+ and GFAP+ populations, Kruskal–Wallis and Dunn's multiple comparisons test was used. In all cases, comparisons with NT cells were performed. (D, E) Cell viability analysis of patient‐derived GSCs: GBM18 (D) and GBM27 (E) (*n* = 3 for each cell line). Viability values were normalized to their respective DMSO controls (0.2 μL·mL^−1^ media). Statistical differences were determined by comparing each treatment with its DMSO control for the specified time point and drug concentration using a two‐way ANOVA and Dunnett's multiple comparisons test. (F–I) qRT‐PCR analysis of TAp73 (F, H) and CD133 (G, I) expression in GBM18 (F, G) and GBM27 (H, I) cells after 48 h of BMT9 treatment (*n* = 3). Expression values were normalized against the NT cells. One‐way ANOVA and Dunnett's multiple comparisons test were used to address statistical significance (BMT9‐treated cells compared with NT control cells). All the graphs display the mean value ± SEM (standard error of the mean); ns: non‐significant; *P < 0.05, **P < 0.01, ***P < 0.001.

Next, we investigated whether BMT9, like TAp73 deficiency, would also reduce GSC stemness potential. To that extent, we analyzed the expression of stem cell markers CD133 and Nestin and observed that BMT9 was able to reduce CD133 expression at RNA levels (Fig. 6A) with a concomitant decrease in the percentage of CD133+ cells, as well as the mean intensity of this marker (Fig. 6B upper and bottom panel, respectively). Furthermore, although some level of spontaneous differentiation was detected in non‐treated and DMSO‐treated cells, BMT9 induced a significant reduction of Nestin in G144 and G179 cells (Fig. 6C and Fig. S3G, respectively). This decrease in the stemness marker was accompanied by a highly significant increase in the percentage of Tuj1+ and GFAP+ cells after 72 h of treatment under proliferating culture conditions (Fig. 6C), all supporting the idea that this compound reduces the stemness potential of G144 cells.

To corroborate the effect on stemness in other GSC lines and emphasize the clinical implications of our previous findings, we extended our investigations to patient‐derived GSCs‐enriched cultures (GBM18 and GBM27) [47]. These cells have proven to be highly resistant to common GB treatments, especially TMZ. As expected, treatment with the previously used concentrations for TMZ and BCNU (750 and 150 μm, respectively) showed low cytotoxic effects against GBM18 and GBM27 (Fig. 6D,E). On the contrary, the dose–response curve for BMT9 indicated that the two cell lines were susceptible to the novel natural compound (Fig. 6D,E). Interestingly, the GSCs‐enriched lines were even more sensitive than G144 or G179 cells with an IC50 between 1.125 and 2.25 μm at 72 h, which was less than half the IC50 for G144 (4.5 μm). Next, we analyzed TAp73 and CD133 expression in response to BMT9 treatment at sublethal concentrations (0.56 μm). Consistent with our previous results, BMT9 strongly reduced the expression of both TAp73 and CD133 (Fig. 6F–I), indicating that the drug not only reduced cell viability, but also cell stemness, and pointing to BMT9 as a promising therapeutic candidate for GSCs‐enriched tumors.

### 3.6 BMT9 treatment modifies the transcriptional profiles of GSCs and represses gene networks related to proliferation, cell‐cycle progression, and stemness

To further substantiate the effect of BMT9 on stemness and to gain a deeper understanding of the signaling mechanism underlying the effect of BMT9 on GSCs, we performed a transcriptomic analysis by RNA‐seq in G144 cells (Table S3). Principal component analysis showed that the control untreated cells (CTR) and DMSO‐treated samples grouped together, whereas cells treated with BMT9 clearly differentiated from the controls (Fig. S4A). The PC1 component captured 58.46% of the variance, indicating that the treatment clearly affected the transcriptomic profile of these cells. The differential expression analysis supported that the treatment had a large impact on the GSCs transcriptional profile, with 1751 upregulated DEGs and 1772 downregulated genes when compared with CTR, and almost identical results versus DMSO‐treated cells (1737 upregulated and 1745 downregulated genes) (Fig. S4B), while no DEGs were detected when comparing CTR and DMSO (Fig. S4C).

**Fig. 7:**
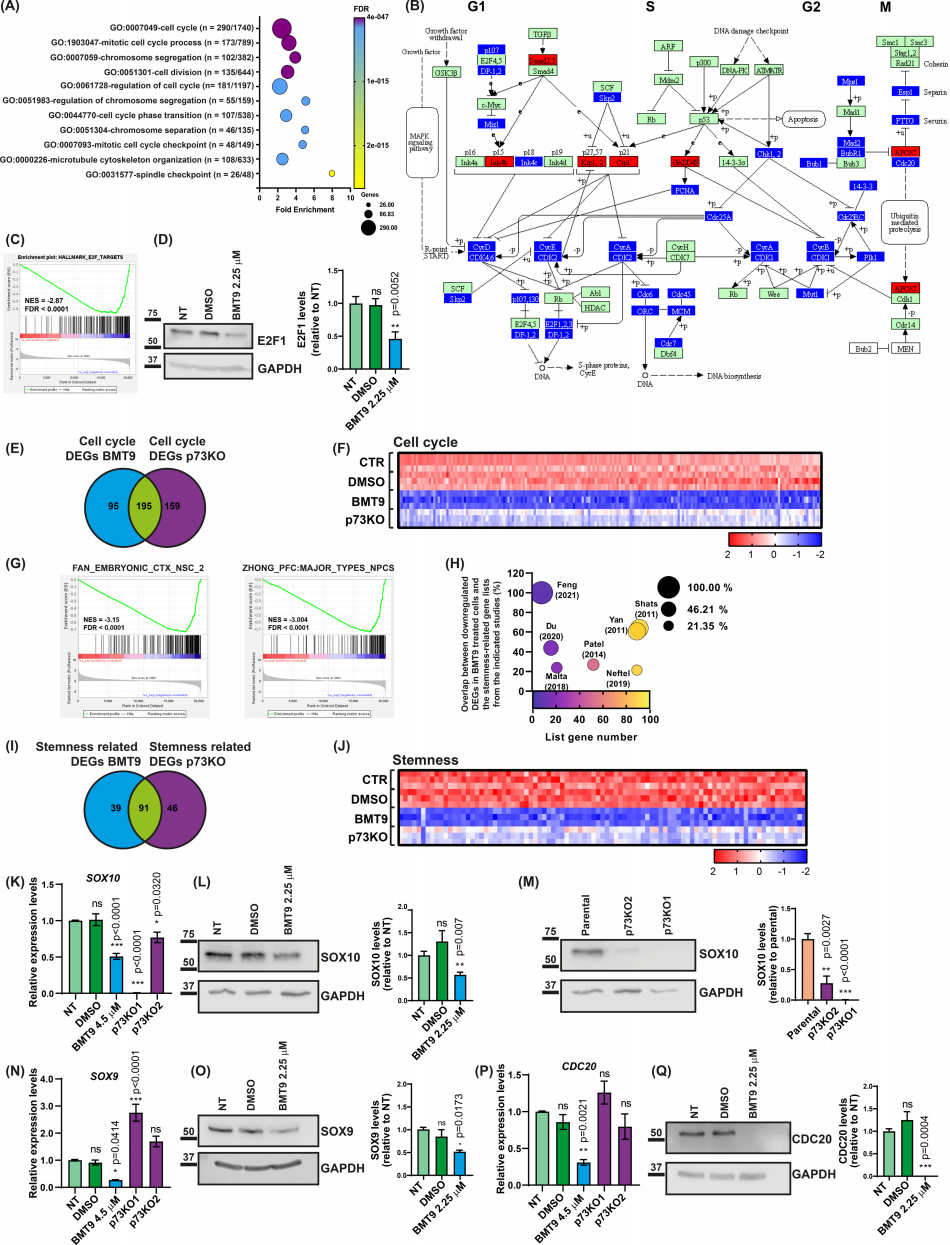
Effect of BMT9 on stem‐like glioblastoma cells transcriptome. G144 cells were treated with 4.5 μm BMT9 for 48 h, and their transcriptome was analyzed by RNA‐seq and compared with untreated (CTR) and DMSO‐treated controls (0.2 μL·mL^−1^ media). Sequencing data were generated from three replicates per condition. (A) Functional annotation analysis of the downregulated differentially expressed genes (DEGs) (*P*‐adj < 0.01) using the Gene Ontology (GO) Biological Process (BP) dataset. The top statistically significant terms with at least a twofold enrichment are displayed. (B) KEGG chart of the ‘Cell cycle pathway’ (hsa04110), highlighting the upregulated (red) and downregulated (blue) DEGs by BMT9 treatment. (C) Gene Set Enrichment Analysis (GSEA) plot for the Hallmark gene set ‘E2F targets’ that was enriched in BMT9‐treated cells. Normalized enrichment score (NES) and false discovery rate (FDR) are indicated. (D) Analysis of E2F1 protein levels by western blot of G144 cells treated with BMT9 (2.25 μm for 48 h), DMSO (0.2 μL·mL^−1^ media), and non‐treated (NT) controls (*n* = 3). Protein quantifications were normalized to their respective loading control (GAPDH), and relative values to the NT cells are represented. Statistical significance (versus NT) was estimated using a one‐way ANOVA with Dunnett's multiple comparisons test; ns: non‐significant. (E) Venn diagram showing the comparison of the downregulated DEGs classified in the BP term ‘GO:0007049‐cell cycle’ after BMT9 treatment and in p73KO cells, highlighting the overlap between both datasets. (F) Heatmap of the 195 cell‐cycle genes commonly downregulated in both BMT9‐treated cells and *TP73* knockout (p73KO) cells. The *Z*‐score expression values (color gradient) were calculated from the FPKM data (fragments per kilobase of transcript per million mapped reads). (G) GSEA plots for stemness‐related genes from the ‘C8: cell type signature gene sets’ that were enriched in BMT9‐treated cells. (H) Bubble plot representing the overlap between the downregulated DEGs in G144 cells treated with BMT9 and the stemness‐related gene lists from the indicated studies. Horizontal axis and color gradient show the number of genes included in each study. Vertical axis and bubble size illustrate the percentage of overlap between each study and our downregulated DEG list. (I) Venn diagram showing the overlap between the stemness‐related genes downregulated in BMT9‐treated cells and p73KO cells. (J) Heatmap representing the *Z*‐score expression values of the 91 stemness‐related genes commonly downregulated by BMT9 treatment and p73 inactivation. (K–Q) Expression analysis of the indicated stemness‐related genes by qRT‐PCR (K, N, P) and western blot (L, M, O, Q) in p73KO1 and p73KO2 cells, as well as parental G144 cells treated for 48 h with the indicated concentration of BMT9, or DMSO and NT controls (*n* = 3). GAPDH was used as loading control. Values were normalized and statistically compared with the NT cells (K, L, N, O, P, Q) or to the parental cells (M). Statistical differences were determined using a one‐way ANOVA with Tukey's multiple comparisons test (K, N, O, P, Q) or unpaired *t*‐test (L, M). All the graphs display the mean value ± SEM (standard error of the mean); ns, non‐significant; *P < 0.05, **P < 0.01, ***P < 0.001.

To identify which biological processes were most affected by BMT9 treatment, we performed a functional annotation analysis using the common downregulated DEGs from the deseq2 comparisons: BMT9 vs CTR and BMT9 vs DMSO (see Venn diagram in Fig. S4D). The common gene list (1609 downregulated DEGs) was analyzed using DAVID. In consonance with our previous functional assays, in which BMT9 impaired GSCs cell growth and stemness, the most statistically significant GO terms, with at least a two‐fold enrichment, referred to GO Biological Processes such as ‘cell division’ and ‘cell cycle progression’ (Fig. 7A), resembling our data from the transcriptomic analysis of p73KO cells. Reactome database analysis and GSEA (Fig. S4E) further confirmed these results, with the ‘R‐HSA‐1640170 ~ Cell Cycle’ term having the highest significance (FDR 6.39 × 10^−49^; fold enrichment = 3.29; 184 genes included). The comparison of our whole DEGs list with the KEGG database ‘Cell Cycle pathway’ (hsa04110; https://www.genome.jp/pathway/hsa04110) illustrated that BMT9 was repressing some of the main regulators of cell‐cycle progression, like genes from the E2F1 family, different cyclins (cyclin D, E, A, and B), and cyclin‐dependent kinases like CDK4 (Fig. 7B and Table S4). As in the case of p73 inactivation (Fig. 2E), GSEA analysis showed downregulation of E2F1 targets and E2F1 signaling signature (Fig. 7C). qRT‐PCR and western blot analysis confirmed that E2F1 levels were strongly downregulated upon BMT9 treatment (Fig. S4F and Fig. 7D, respectively). Moreover, negative regulators of cell cycle, such as *CDKN1A* (p21), *CDKN1B* (p27), and *CDKN2B* (INK4B), were upregulated by the treatment (Fig. 7B, Fig. S4G and Table S4). To further address whether the proliferation‐related genes repressed by BMT9 overlap with the set of p73‐related proliferation genes that we had previously identified, we compared the downregulated DEGs classified in the GO term cell cycle (GO:0007049 ~ cell cycle), which was the most enriched BP term after BMT9 treatment and in p73KO cells (Fig. 7E and Table S6). We found that 195 genes were commonly downregulated (67% of the DEGs repressed by BMT9), supporting the idea that part of the effect of BMT9 on cell proliferation could be associated with TAp73 deficiency. Nonetheless, the impact of BMT9 treatment on cell‐cycle transcriptional programs was stronger (Fig. 7F), suggesting that BMT9 could repress GSCs proliferation through additional mechanisms other than TAp73 repression.

We next asked whether transcriptional signatures linked to GSC stemness were affected by BMT9 treatment. GSEA analysis revealed that gene signatures related to NSCs [69] and neural progenitor cells [70] were negatively affected by BMT9 treatment, supporting the idea that BMT9 reduces NSC identity in GSCs (Fig. 7G). Thus, we compared the previously identified GSCs gene signatures (Table S5) with our DEGs list. Strikingly, a high percentage of genes from the lists were common to our downregulated DEGs after BMT9 treatment (Fig. 7H) and, as observed with the p73KO cells, the higher number of overlapping genes coincides with the gene signatures from Shats et al. [76] (58/91) and Yan et al. [77] (54/89), which mostly represent the transcriptome profile associated with CD133+ GSCs, altogether strongly supporting our functional data that revealed BMT9 specificity against CD133+ GSCs and stemness repression. Of note, it was the complete match of the gene list from Feng et al. [72] with our DEGs. These authors proposed eight candidate ‘stemness genes’ in CSCs from head and neck squamous cell carcinoma, including the cyclin‐dependent kinase inhibitor 3 (*CDKN3*), several kinesin family members (*KIF14*, *KIF18A* and *KIF23*), DLG‐associated protein 5 (*DLGAP5*) involved in the progress of centrosome‐independent mitotic spindle assembly, the TKK protein kinase (*TTK*), the cytoskeleton‐associated protein 2 like (*CKAP2L*), and *BUB1* mitotic checkpoint serine/threonine kinase (*BUB1*). Since most of these genes are related to mitotic activity and chromosome segregation [72], the fact that they are downregulated by the compound (log_2_ fold changes up to −2.73 in the case of *KIF14* or *BUB1* and −2.96 for *DLGAP5*) reinforces our results on the impact of BMT9 treatment on cell cycle. Interestingly, five of these ‘candidate stemness genes’ (*BUB1*, *CKAP2L*, *KIF14*, *KIF23*, and *TTK*; Table S5) are also downregulated in the knockout line, supporting the idea that the compound could be exerting its function, at least in part, through the downregulation of *TP73*.

To elucidate to which extent p73 was the mediator of BMT9 effect, we compared the effects of BMT9 treatment versus p73‐deficiency on stemness gene expression. To this purpose, we generated a consensus gene list of 342 genes by integrating the GSC stemness‐associated gene lists from the studies used before (Table S5) with other genes reported to be associated with GSC stemness [88, 89, 90, 91]. Noticeably, around 40% of the GSC stemness genes were downregulated by p73 elimination and by BMT9 treatment (137/342 and 130/342, respectively), from which 51% (91/176) were downregulated in both BMT9‐treated cells and p73KO cells (Fig. 7I,J). An example of the commonly downregulated genes at RNA and protein levels are *CD133* (Figs 1F,G and 6A,B), and *SOX10* (Fig. 7K–M). BMT9 treatment led to a higher repression of stemness genes than p73 inactivation alone (Fig. 7J and Tables S3 and S6) and some GSC stemness‐related markers were only downregulated by BMT9. These results indicate that the effect of BMT9 on GSC stemness could be partially, but not totally, caused by TAp73 repression; thus, BMT9 exerts stemness repression by p73‐dependent and independent mechanisms. An example of genes downregulated only by BMT9 are *SOX9*, essential for GSC self‐renewal [92], and *CDC20* which is required for invasiveness, self‐renewal, and *in vivo* tumorigenicity of GSCs [90] (Fig. 7N–Q). Importantly, they were consistently downregulated by BMT9 in three different GSC cell lines (Fig. S4H), altogether supporting BMT9 as a promising candidate therapeutic agent against GB.

## 4 Conclusions

The role of the p53 family in tumor progression has been well studied in the cancer field. An initial paradigm was proposed based on data regarding TAp73 structural similarities with p53 and its capacity to transactivate p53‐related genes that regulate cell‐cycle arrest and apoptosis induction [93, 94], as well as the reported *in vivo* data [95, 96, 97, 98]. This paradigm postulated that *TP73* had a bimodal function where TAp73 would exert a p53‐like function acting as a tumor suppressor gene, while DNp73 could have a pro‐oncogenic role. However, the compiled data up to date have brought up a more complex picture which proposes that TAp73 could function in early tumor stages as a tumor suppressor by regulating apoptosis in response to genotoxic stress while, at advance stages, it could favor tumor progression [99]. This paradigm is supported by the high TAp73 levels detected in multiple tumor types [100].

TAp73 is a key regulator of murine neurogenesis as it is crucial for the maintenance of an undifferentiated pool of NSC and for their correct differentiation into fully mature neurons [101]. In line with TAp73 function in NSC maintenance, its role in brain tumors is of great interest, since the accumulated data highlights TAp73 as a fundamental factor in GB progression and invasion and as a positive regulator of the growth and stemness of GSCs [14, 81], which postulates TAp73 as a putative therapeutic target. In this work, we show that the elimination of p73 in GSCs promotes their differentiation, similar to the effect observed on murine NSCs. Moreover, we demonstrated that p73 and, specifically the TAp73 isoform, sustains GSC self‐renewal, being a central regulator of transcriptional networks related to the stemness signature of CSCs, supporting previous reports that postulated that TAp73 positively regulates growth and self‐renewal of CD133+ patient‐derived brain tumor‐initiating cells [14].

All this evidence gave us a solid biological rationale to propose that targeting strategies against TP73 should be considered when developing new anticancer therapeutic strategies. Thus, as a proof of concept, we identified a novel natural compound, BMT9, capable of reducing TAp73 expression in GSC and GB cells and tested its effect on their viability, cell proliferation, and stemness. BMT9 was more effective *in vitro* than first‐line drugs TMZ and BCNU and showed specificity against CD133+ GSCs. More interestingly, BMT9 treatment reduced GSC stemness potential, migration, and invasiveness. Nonetheless, the impact of BMT9 treatment on cell cycle and GSC stemness transcriptional programs was stronger than the elimination of p73 alone, indicating that BMT9 acts by p73‐dependent and independent mechanisms.

This tumor‐promoting role of *TP73* would fit with the recently proposed model of metastasis evolution in which the co‐option of developmental programs increases metastatic potential [102, 103]. In this regard, hijacking of *TP73*‐regulated neurodevelopmental programs, including neural stemness, in TAp73‐expressing tumors, might increase tumor progression and metastatic competence [104]. This hypothesis will concur with recent data demonstrating that DNp63/p73 axis drive metastatic colonization by controlling a regenerative epithelial stem cell program in quasi‐mesenchymal CSCs [12]. This co‐option process would imply the reactivation of the *TP73* gene outside its physiological context, resulting in the TAp73 overexpression detected in advanced stages of tumors [99]. However, the nature of the mechanisms that cause this reactivation remains elusive. Multiple factors might impinge in TAp73 overexpression in cancer cells, including E2F1‐mediated regulation of TAp73 transcription [57], especially in the context of p53‐inactivation [105], or TAp73 stabilization under hypoxic conditions via HIF1‐α [106], among other possible mechanisms. Furthermore, it is very likely that TAp73 overexpression would be sustaining cell growth at several levels. For example, TAp73 plays a key role in the activation of AP‐1 target genes, and the absence of p73 compromised their induction [34]. This AP‐1 attenuation could hinder E2F1 expression and cause a decrease in the E2F1‐upregulated cell‐cycle genes [107]. In agreement with this effect, our RNA‐seq analysis revealed downregulated levels of E2F1, as well as E2F1 targets, upon p73 elimination in p73KO cells and after BMT9 treatment. This scenario would suggest the existence of a feedback loop between E2F1 and TAp73 in cancer cells, that would be short‐circuited by BMT9 treatment. Even though further studies are needed to validate BMT9 effectiveness *in vivo*, overall our data support this novel natural compound as a promising candidate therapeutic agent against GB, opening the door to alternative chemotherapeutic solutions that could be of great relevance for patients.

## Conflict of interest

During this study, Dr M. Martín‐López, Dr J. M. Sanchez, and Dr A. Fernandez were supported by Biomar Microbial Technologies.

## Author contributions

JV‐F contributed to the investigation, conceptualization, methodology, validation, formal analysis, visualization, and writing—original draft. NM‐G contributed to the investigation, methodology, validation, and visualization. MM‐L, AV‐J, and LL‐F contributed to the investigation and methodology. LM‐A contributed to the software, data curation, investigation, and methodology. LM‐H, NG‐R, and JMS contributed to the investigation, methodology, and resources. AF and AA‐S contributed to the resources, funding acquisition, and supervision. MMM contributed to the conceptualization, formal analysis, software, data curation, visualization, writing—original draft, project administration, and supervision. MCM contributed to the conceptualization, writing—original draft, funding acquisition, project administration, and supervision. All the authors contributed to the drafting of the manuscript, and writing—review and editing.

## Supporting information


**Fig. S1.** Characterization of p73 inactivation in G144 GSCs.
**Fig. S2.** Validation of the LV‐P1‐TP73‐Fluc‐DsRed2 reporter vector system.
**Fig. S3.** Effect of BMT9 treatment on G179 GSC cells.
**Fig. S4.** Analysis of the transcriptomic data of BMT9‐treated G144 cells.

**Table S1.** Primary and secondary antibodies.
**Table S2.** Primer sequences for qRT‐PCR analysis.

**Table S3.** RNA sequencing data (FKPMs) from G144‐p73KO cells and control cells (CTR).

**Table S4.** Differentially expressed genes (DEGs) in G144‐p73KO cells and BMT9‐treated cells assigned to the KEGG database ‘Cell Cycle pathway’.

**Table S5.** Stemness‐related genes from published signatures that are downregulated DEGs in G144‐p73KO2 cells.

**Table S6.** Differential expression analysis comparing BMT9‐treated cells versus control (CTR) or DMSO‐treated cells. List of DEGs used to generate the Cell cycle and Stemness heatmaps.

**Video S1.** Cell morphology of G144 parental cells.

**Video S2.** Cell morphology of G144 p73KO2 cells.

## Data Availability

The lists of antibodies and primers, as well as the supplementary data that supports the findings of this study, are available in the supplementary tables of this article (Tables [Link], [Link]). The BMT9 compound is commercially available at https://biomarmt.com/productos/.
