# Supplementary material for: A novel TAp73‐inhibitory compound counteracts stemness features of glioblastoma stem cells
Source: Mol Oncol. 2024 Aug 1;19(3):852–77. doi: 10.1002/1878-0261.13694 (PMC11887682; doi:10.1002/1878-0261.13694)
Supplement: Supplementary file 1 — Fig. S1. Characterization of p73 inactivation in G144 GSCs. Fig. S2. Validation of the LV‐P1‐TP73‐Fluc‐DsRed2 reporter vector system. Fig. S3. Effect of BMT9 treatment on G179 GSC cells. Fig. S4. Analysis of the transcriptomic data of BMT9‐treated G144 cells. [file MOL2-19-852-s001.zip › Supplementary_Figure_legends.docx]

**Supplementary figures**

**Fig. S1.** **Characterization of p73 inactivation in G144 stem-like glioblastoma cells (GSCs).** (A) Sequencing electropherograms of the G144-p73KO clones and pairwise alignment of the targeted region of both p73KO clones and the corresponding sequence of the wild-type (WT) *TP73* gene. The orange box marks the sgRNA target sequence, and the red arrow indicates the Cas9 nuclease cleavage site. (B) Aminoacidic sequence at the target site and downstream amino acid sequence resulting from the *TP73* WT and edited sequences. The red arrow indicates the adenine insertion site and the black arrow points the location where the edited sequence originates a STOP codon. (C) qRT-PCR analysis confirming the inactivation of the *TRP73* gene in the p73KO selected clones using primers that match the Cas9 target sequence (n=3). (D) Western blot analysis of the selected clones using a TAp73 specific antibody (n=3). GAPDH was used as the loading control. (E) Representative phase contrast images of the G144 parental cells and the selected clones. Scale bar: 250 µm. (F) Representative phase contrast images of the polar and apolar morphology of G144 cells. Scale bar: 50 µm. The graph represents the percentage of cells from each category for the indicated cell lines. At least 70 cells per condition were quantified in three independent experiments. To test the association between the genotype and the categorical variable, a contingency table analysis was used followed by Fisher’s exact test. ns=non-significant; ***p<0.001.

**Fig. S2.** **Validation of the LV-P1-*TP73*-Fluc-DsRed2 reporter vector system.** (A) Fluorescence microscopy images of different human cell lines transfected with the construct (n=3). Scale bar: 200 µm. (B) TAp73 RNA expression in HCT116 p53-/- cells treated with Doxorubicin (1 µM) for 12, 24 and 48 h (n=3). One-way ANOVA was used to estimate statistical differences relative to the initial time point (0 h). ** p<0.01. (C) Analysis of TAp73 protein levels by western blot in HCT116 p53-/- cells treated with Doxorubicin (1 µM) for 12 and 24 h and non-treated controls (NT) (n=3). TAp73α and TAp73β controls were included as indicated.

**Fig. S3.** **Effect of BMT9 treatment on G179 stem-like glioblastoma cells.** (A) TAp73 RNA expression analysis in G179 cells treated with the indicated concentrations of BMT9 or DMSO (0.2 µl/ml media) (n=3). Expression was normalized to the non-treated cells (NT) expression value. One-way ANOVA followed by Dunnett’s multiple comparisons test was used to calculate statistical differences (versus NT cells). (B-D) Analysis of TAp73 (n=7) and phosphorylated p53 (Serine 15) (n=4) protein levels by western blot analysis after 72 h of treatment at the indicated doses. Protein quantifications were normalized to their respective loading control (GAPDH), and relative values to the NT cells are represented (C and D). Unpaired t-tests were performed to determine the statistical significance (comparison to NT cells). (E, F) Analysis of BMT9 effect on migration and invasion using the wound healing (E) (n=4) and 3D invasion assay (F) (n=3), respectively. DMSO: 0.2 µl/ml media. Quantified areas are marked in green. Scale bar: 250 µm (E) and 100 µm (F). (G) Immunofluorescence of G179 cells under proliferating conditions treated with BMT9 for 72 h (or the corresponding NT and DMSO controls; 0.2 µl/ml media), using anti-Nestin (red) antibody (n=3). Nuclei were counterstained with DAPI. Scale bar: 20 μm. The graph on the right corresponds to the quantification of the Nestin mean fluorescence intensity (MFI). A minimum of 350 cells per condition were analyzed. Statistical differences were calculated using a one-way ANOVA (comparison to NT cells). All the graphs display the mean value ± SEM (standard error of the mean); ns=non-significant; *p<0.05, ** p<0.01, ***p<0.001.

**Fig. S4. Analysis of the transcriptomic data of BMT9-treated G144 cells.** (A) Principal Component analysis of the three replicates per condition: untreated cells (CTR), DMSO treated cells (0.2 µl/ml), and BMT9 treated cells (4.5 µM, TTO). (B) Volcano plots indicating the Differentially Expressed Genes (DEGs) between BMT9 treated cells versus CTR and DMSO treated cells. Red and blue dots depict the upregulated and downregulated genes in BMT9 treated cells, respectively. (C) Volcano plot representing that no differentially expressed genes were detected between CTR and DMSO treated cells. (D) Venn diagram representing the DEGs from (B). The intersection indicates the common DEGs in both comparisons. (E) Gene Set Enrichment Analysis (GSEA) revealed that gene sets related with cell cycle progression were downregulated by BMT9 treatment. GSEA plots for selected gene sets from the indicated Molecular Signatures Databases (“C5: ontology gene sets”, “H: Hallmark Gene sets” and “C2: curated gene sets'') are shown. Normalized enrichment score (NES) and False Discovery Rate (FDR) are indicated. (F) RNA expression levels of E2F1 in G144 cells after 48 h of BMT9 treatment (4.5 µM). Data was normalized against the non-treated (NT) control cells. (G) FPKM values of selected negative cell cycle regulators in BMT9-treated G144 cells, and its corresponding untreated (CTR) and DMSO treated controls. Statistical differences versus the DMSO controls were determined using an unpaired t-test. (H) RNA expression levels of the stemness markers SOX9 and CDC20 in stem-like glioblastoma cells (GSCs) G179 and patient-derived GSCs-enriched lines GBM18 and GBM27. Cells were treated with the indicated BMT9 concentrations for 72 hours (G179) or 48 hours (GBM18, GBM27). All the graphs display the mean value ± SEM (standard error of the mean); ns=non-significant; *p<0.05, ** p<0.01, ***p<0.001.
