# Supplementary material for: A novel TAp73‐inhibitory compound counteracts stemness features of glioblastoma stem cells
Source: Mol Oncol. 2024 Aug 1;19(3):852–77. doi: 10.1002/1878-0261.13694 (PMC11887682; doi:10.1002/1878-0261.13694)
Supplement: Supplementary file 1 — Fig. S1. Characterization of p73 inactivation in G144 GSCs. Fig. S2. Validation of the LV‐P1‐TP73‐Fluc‐DsRed2 reporter vector system. Fig. S3. Effect of BMT9 treatment on G179 GSC cells. Fig. S4. Analysis of the transcriptomic data of BMT9‐treated G144 cells. [file mol213694-FigS1.pdf › mol213694-FigS1.pdf]

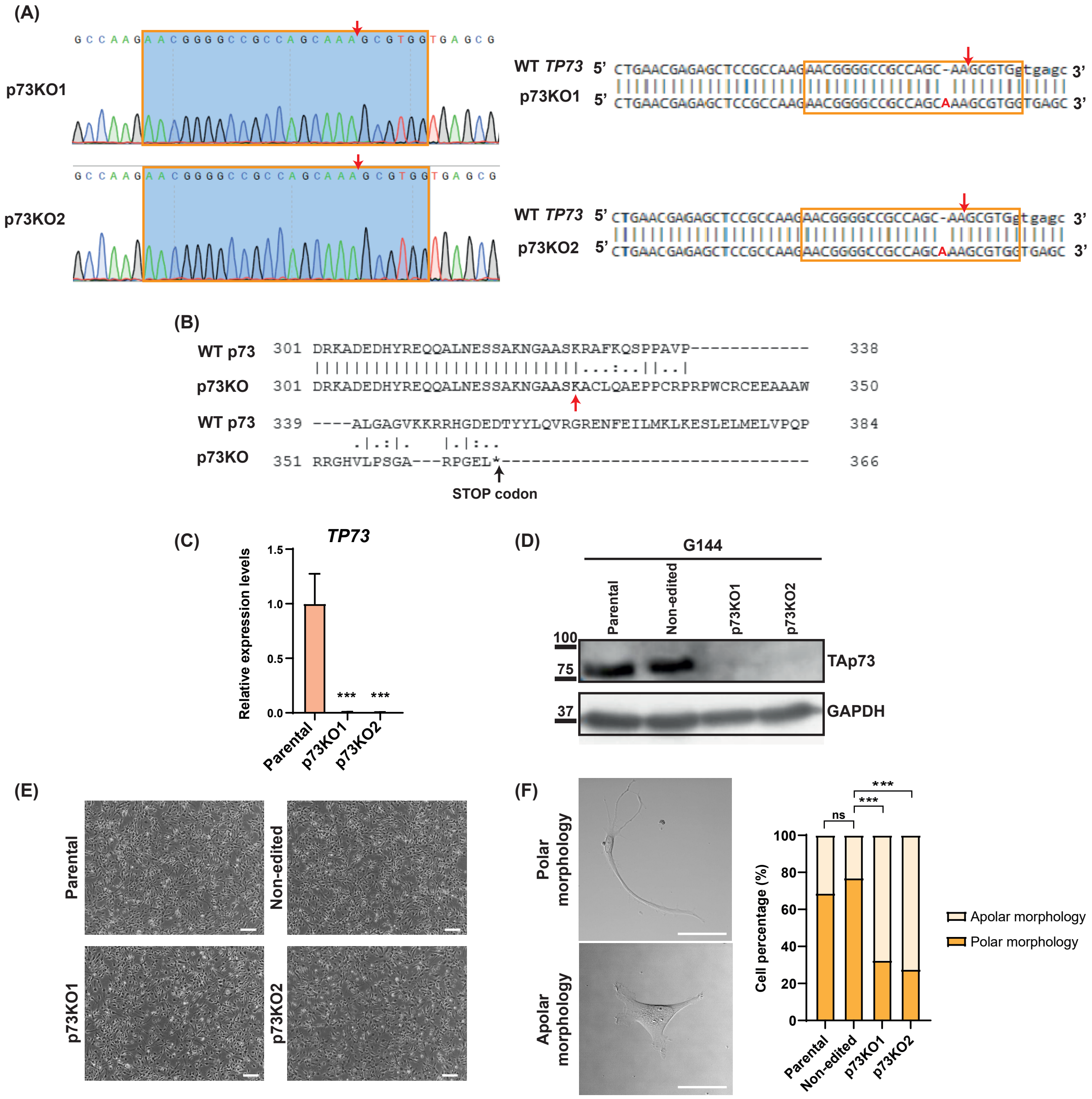

Villoch *et al.*, Fig. S1
