# Supplementary material for: A novel TAp73‐inhibitory compound counteracts stemness features of glioblastoma stem cells
Source: Mol Oncol. 2024 Aug 1;19(3):852–77. doi: 10.1002/1878-0261.13694 (PMC11887682; doi:10.1002/1878-0261.13694)
Supplement: Supplementary file 5 — Table S5. Stemness‐related genes from published signatures that are downregulated DEGs in G144‐p73KO2 cells. [file MOL2-19-852-s003.pdf]

Table S5. Stemness-related genes from published signatures that are downregulated DEGs in G144-p73KO2 cells

**Stemness signatures**		**Stemness-related genes**
**Shats *et al.* (2011)**	This work defines a Consensus Stemness Ranking (CSR) signature upregulated in cancer stem cell-enriched samples at advanced tumor stages. The capacity of the CSR signature to detect stem-like characteristics was tested with CD133+ and CD133– cells from two GB xenograft tumors. Microarray profiling demonstrated that the CSR signature was high in the CD133+ population	*ASPM, ATAD2, BIRC5, BUB1, BUB1B, CCNB1, CCNB2, CDC20, CDC7, CENPF, CKS1B, CST3, DBF4, DTL, ESPL1, FBXO5, FEN1, FOXM1, GPSM2, HMGB2, IGFBP4, KIF11, KIF14, KIF15, KIF23, KIF2C, KIF4A, MAD2L1, MCM4, MCM6, MELK, NCAPG, NCAPH, PAICS, PBK, PTTG1, PUS7, RACGAP1, RAD51, RAD51AP1, RCC1, RFC4, RRM2, SMC2, SNRPA1, TOP2A, TPX2, TRIP13, TTK, UBE2C, ZWINT*
**Yan *et al.* (2011)**	Transcriptomic profiling of CD133+ and CD133– by microarrays from 5 fresh patient-derived tumor samples allowed the definition of a CD133 signature (214 DEGs) resembling the expression profile of human embryonic stem cells and *in vitro* cultured GSCs. This signature contains a subset of 89 transcripts upregulated in the CD133+ population	*ARHGAP11A, ASPM, BIRC5, BRCA1, CENPH, CENPK, CKAP2L, CTNNAL1, DHFR, DIAPH3, DTL, ENAH, FANCI, FBXO5, GINS2, GMNN, HMGB2, JAM2, KIF11, KIF15, KIF2C, KIF4A, KNTC1, LIG1, MAD2L1, MCM2, MCM3, MELK, MND1, NCAPH, NUF2, PBK, PCNA, POLQ, PRIM1, PTTG1, RAD51, RANBP1, RRM2, SMC2, SNRPE, TIMELESS, TM4SF1, TOP2A, TPX2, TRIP13, TTK, WDR34*
**Patel *et al.* (2014)**	Using single-cell RNA-seq data from 5 primary GB samples, they defined a stemness signature that is inversely correlated with cell cycle signature	*ATP1B2, CALM1, CCND2, FABP7, FDFT1, FXYD6, GAP43, GPM6A, GRIA2, HAS2, ID1, MAGED1, MAP2, MARCKS, PMP2, PTPRZ1, RUFY3, SEC11C*
**Malta *et al.* (2018)**	This study compares multiple, publicly available, molecular profiles from cells that exhibit various degrees of stemness and uses machine learning to define indices to quantify stemness. Higher values for these indices were associated with biological processes active in CSCs and with tumor dedifferentiation reflected in histopathological grade	*EZH2, HIF1A, LGR5, MYC, NOTCH1*
**Neftel *et al.* (2019)**	Using RNA-seq data from 20 adult and 8 pediatric GB samples, this work identifies several transcriptional meta-modules, like the stem and progenitor cell signatures, which also includes neural progenitor markers	*ABAT, ASCL1, BCAN, DLL1, DLL3, ELAVL4, ELMO1, FXYD6, GLCCI1, GRIK2, HES6, KIF5A, LRRN1, MAP2, MARCKSL1, NEU4, OLIG1, PKIA, SEZ6L, SHD, SOX4, STMN4*
**Du *et al.* (2020)**	Based on the results from Malta *et al.* (2018), these authors propose a subset of 18 genes highly related with stemness in GB. Knockdown of this gene set inhibited tumor growth in a xenograft model	*AURKA, DBF4, NUF2, RPA3, RBMX, RRM2, TMEM97, TPX2*
**Feng *et al.* (2021)**	Using a machine learning approach, they select a gene list and use it to calculate a stemness index in 44 samples of head and neck squamous cell carcinoma (and their healthy counterparts)	*BUB1, CKAP2L, KIF14, KIF23, TTK*
