# Supplementary material for: A novel TAp73‐inhibitory compound counteracts stemness features of glioblastoma stem cells
Source: Mol Oncol. 2024 Aug 1;19(3):852–77. doi: 10.1002/1878-0261.13694 (PMC11887682; doi:10.1002/1878-0261.13694)
Supplement: Supplementary file 2 — Table S1. Primary and secondary antibodies. Table S2. Primer sequences for qRT‐PCR analysis. [file MOL2-19-852-s004.pdf]

Table S1. Primary and secondary antibodies. IF: Immunofluorescence. WB: Western blot.

	Antibody	Host	Dilution	Manufacturer	Catalog Nº	Use	Membrane	Incubation
Primary	GFAP	Rabbit	1:200	NeoMarkers	RB087A1	IF	-	O/N 4 ºC
	Nestin	Mouse	1:200	BD Bioscience	611658	IF	-	O/N 4 ºC
	Phospho-His3 (Ser10)	Mouse	1:200	Cell Signaling	9706s	IF	-	O/N 4 ºC
	Tuj1	Mouse	1:1000	Covance	801201	IF	-	O/N 4 ºC
	Actin	Rabbit	1:10000	Sigma-Aldrich	A5060	WB	Nitrocellulose	O/N 4 ºC
	CDC20	Rabbit	1:1000	Proteintech	10252-1-AP	WB	PVDF	1.5 h RT
	E2F1 (KH95)	Mouse	1:1000	Santa Cruz	sc-251	WB	PVDF	1.5 h RT
	GAPDH	Mouse	1:25000	Abcam	Ab8245	WB	Nitrocellulose	O/N 4 ºC
	p21 (C-19)	Rabbit	1:500	Santa Cruz	sc-397	WB	PVDF	1.5 h RT
	Pan-p73	Rabbit	1:1000	Abcam	Ab40658	WB	Nitrocellulose	O/N 4 ºC
	Phospho-Histone H2A.X (Ser139)	Rabbit	1:500	Proteintech	29380-1-AP	WB	PVDF	1.5 h RT
	Phospho-p53 (Ser15)	Rabbit	1:1000	Cell Signaling	9284	WB	Nitrocellulose	O/N 4 ºC
	SOX9	Mouse	1:1000	Proteintech	67439-1-Ig	WB	PVDF	1.5 h RT
	SOX10	Mouse	1:1000	Proteintech	66786-1-Ig	WB	PVDF	1.5 h RT
	TAp73	Rabbit	1:2000	Abcam	ab14430	WB	Nitrocellulose	O/N 4 ºC
Secondary	Alexa Fluor 488 anti-mouse IgG2a	Goat	1:1000	Molecular Probes	A21131	IF	-	45 min RT
	Alexa Fluor 594 anti-mouse IgG1	Goat	1:1000	Molecular Probes	A21125	IF	-	45 min RT
	Alexa Fluor 647 anti-rabbit IgG	Goat	1:1000	Molecular Probes	A21244	IF	-	45 min RT
	Alexa Fluor 488 anti-mouse IgG	Donkey	1:1000	Molecular Probes	A21202	IF	-	45 min RT
	Anti-Rabbit IgG (H+L) HRP	Goat	1:20000	Thermo Fisher Scientific	31460	WB	According to primary	45 min RT
	Anti-Mouse IgG (H+L) HRP	Goat	1:20000	Thermo Fisher Scientific	31430	WB	According to primary	45 min RT

Table S2. Primer sequences for qRT-PCR analysis.

Primer	Specie	Forward 5' => 3'	Reverse 5' => 3'
18S	Human	GGCGCCCCCTCGATGCTCTTA	GCTCGGCCTGCTTTGAACAC
TAp73	Human	GCACCACGTTTGAGCACCTCT	GCAGATTAGAACTGGGCATGA
CD133	Human	GAGTCGGAAACTGGCAGATAGCA	ACGCCTTGTCCTTGGTAGTGTTG
OLIG2	Human	TGCGCAAGCTTTCCAAGAT	CAGCGAGTTGGTGAGCATGA
CDC20	Human	GACCACTCCTAGCAAACCTGG	GGGCGTCTGGCTGTTTTCA
FOXM1	Human	ATACGTGGATTGAGGACCACT	TCCAATGTCAAGTAGCGGTTG
SOX9	Human	AGCGAACGCACATCAAGAC	CTGTAGGCGATCTGTTGGGG
SOX10	Human	CCTCACAGATCGCCTACACC	CATATAGGAGAAGGCCGAGTAGA
